# Smallholders’ perceptions on biosecurity and disease control in relation to African swine fever in an endemically infected area in Northern Uganda

**DOI:** 10.1186/s12917-019-2005-7

**Published:** 2019-08-05

**Authors:** Erika Chenais, Susanna Sternberg Lewerin, Sofia Boqvist, Karl Ståhl, Solomon Alike, Bruce Nokorach, Ulf Emanuelson

**Affiliations:** 1 National Veterinary Institute, SVA, ESS, S-75189 Uppsala, Sweden; 2 Swedish University of Agricultural Sciences, Uppsala, Sweden; 3 Veterinary Sector, Production and Marketing Department, Omoro District Local Government, Gulu, Uganda; 4 Veterinary Sector, Production and Marketing Department, Amuru District Local Government, Gulu, Uganda

**Keywords:** Epizootic pig diseases, Prevention, Implementation, Intervention, Smallholders

## Abstract

**Background:**

In Africa, intensified pig production is frequently accompanied by increased occurrence of African swine fever (ASF) outbreaks, leading to high case fatality rates and socio-economic impact for the farmers. ASF control relies on prevention of disease transmission and control of outbreaks. The aim of this study was to increase the understanding on how the knowledge of ASF epidemiology and control can be transferred into successfully implemented biosecurity interventions on farm and community level. Structured interviews with 200 randomly selected, pig-keeping households in northern Uganda were undertaken three times. Perceptions related to general biosecurity and hypothetical control interventions and attitudes towards pig farming were investigated by measuring the agreement to statements using a Likert scale.

**Results:**

Respondents generally conveyed positivism towards pig farming, biosecurity, and the potential of biosecurity for preventing ASF outbreaks. These positive attitudes, as well as the will to invest in biosecurity, were reduced in households that had experienced ASF outbreaks. Among the control interventions change of boots before entering the pig stable was highly accepted and seasonal adaptation of pig rearing times accepted on medium level. Statements on preventive sales of healthy pigs in connection with outbreaks and on buying pork products from slaughter operations receiving ASF-contact pigs received low acceptance, increasing, however, for households that had experienced ASF outbreaks. Consumption of pork from ASF infected pigs was generally not accepted, medium level of agreement was expressed for statements on the zoonotic potential of ASF and for neutralizing ASF by cooking.

**Conclusions:**

To gain in-depth understanding of the complexity of people’s behaviour, reasoning and decision-making processes, deeper involvement of the social sciences and a qualitative research approach might be used for further studies. Communicating information regarding the ASF not being zoonotic, and how the virus is neutralized will be important for increasing acceptance and enhancing implementation for the hypothetical control interventions preventive sales, safe slaughter, and consumption of processed and safe pork. Likewise, participatory development to adopt any control interventions to the local context on community level will be necessary for successful implementation.

**Electronic supplementary material:**

The online version of this article (10.1186/s12917-019-2005-7) contains supplementary material, which is available to authorized users.

## Background

Pig production has been presented as a tool for income generation and poverty reduction for smallholder farmers [1–3]. Pork further adds valuable protein to the diet of many poor [4]. Unfortunately, in Africa, intensified pig production is frequently accompanied by increased occurrence of African swine fever (ASF) outbreaks [5]. ASF is a transboundary disease, caused by a DNA virus within the *Asfarviridae* family, genus *Asfivirus,* that affects pigs with high case fatality rates and related socio-economic impact for the farmers [6]. In Uganda, a low-income county in east Africa, ASF is endemic [7]. There is currently no effective vaccine or treatment available for ASF, thus disease control relies on prevention of disease transmission and rapid control of outbreaks [5, 8]. In viremic animals the virus is present in all bodily secretions [9], although with highest concentrations in blood [10]. The virus is further very resistant if protected by organic material [11–14], but neutralized by heat-treatment, or by standard disinfectants if un-protected [15]. In endemic situation in Africa the virus is mostly transmitted via direct contact between domestic pigs, or via infected blood or pig products [6, 8, 16]. The transmissibility, often measured as R_0_, is further relatively low, especially between farms [17, 18]. This means that disease transmission could be reduced with improved biosecurity on and between farms, and in value chain compartments such as trade and slaughter [6, 8]. We have previously investigated some attitudes to, and consequences of, failed biosecurity [19, 20]. Results from our previous studies [19, 20], from other relevant ASF research [21], as well as from general knowledge on participatory actions and implementation science, all underline the importance of local acceptance and adoption of biosecurity in order to achieve effective implementation [22, 23].

The aim of this study was to increase the understanding on how the current knowledge of ASF control and biosecurity can be transferred into successfully implemented biosecurity measures on farm and community level. More specifically, we wanted to investigate smallholders’ perceptions of biosecurity and control interventions and their attitudes to pig farming. We further wanted to investigate the associations between these perceptions, certain socio-economic factors and outbreaks of ASF, the associations between the perceptions and actions critical for biosecurity, and the variability in perceptions over time.

## Results

Out of the 200 selected households, 198 was reached for the second interview and 196 also the third time. Only the 196 households that participated in all three interview occasions were included in the analyses. For 144 households the same household member responded at the first and second interview, and for 112 households also at the third interview.

### Perceptions on general biosecurity, control interventions, attitudes on pig business and relation with neighbours

In general, a high level of agreement was expressed for statements regarding the preventive potential of biosecurity, see Table 1. Participants also agreed to a high degree with statements pointing out trade as a risk factor. Further, statements regarding pork from ASF infected pigs revealed that participants generally did not want to consume such food items, but were less categorical regarding any associated human health risks. A high level of agreement was expressed for statements concerning the respondents’ possibility to choose trade partners and to tell visitors to not enter the pig stable in their own boots. A low level of agreement was expressed for statements on preventive sales of pigs in connection with outbreaks, and for buying pork from slaughter operations receiving in-contact pigs. According to the results the respondents felt optimistic about their pig farming and also did not report an increase in disagreements in their communities.

**Table 1:** Perceptions on general biosecurity, control interventions, attitudes on pig business and relation with neighbours

Statement	Perception^a^	
	Second occasion^b^	Third Occasion^b^
General biosecurity		
I think it is possible to protect my pigs from getting ASF^c^ by improving farm bio security	4/3.80 (4; 4)	4/3.79 (4; 4)
ASF can not be prevented	2/2.34 (2; 3)	2/2.15 (2; 2)^**^
I would like to invest in farm biosecurity if I received advice on what to do	4/3.79 (4; 4)	4/3.80 (4; 4)
Improved farm biosecurity improves pig health and pig growth	4/3.99 (4; 4)	4/4.09 (4; 4)^**^
Buying live pigs is a risk behaviour for contracting ASF	4/3.87 4; 4)	4/4.10 4; 4)^***^
Frequent selling and buying of pigs is necessary for successful pig farming	2/2.57 (2; 4)	3/2.84 (2; 4)^**^
Eating pork from pigs that have died from ASF is safe for human health	2/2.65 (2; 3)	2/2.65 (2; 3)
I don’t want to eat or buy pork from pigs that have died from ASF	4/4.26 (4; 5)	5/4.43^**^ (4; 5)
Cooking kills the ASF virus	2/2.47 (2; 3)	2/2.63 (2; 4)
If pork prices are lower in the neighbouring village due to them having an outbreak of ASF, I will buy my pork there	2/1.81 (1; 2)	2/1.62 (1; 2)^***^
Control interventions		
If I would get a fair price I would be willing to sell all my healthy pigs when an ASF outbreak occurred in the area	2/2.56 (2; 3)	2/2.42 (2; 3)^*^
I would be happy to buy pork products from a slaughterhouse that receive pigs that have been in contact with pigs dying from ASF	2/1.74 (1; 2)	2/1.67 (1; 2)
I can choose where to/to whom I sell my pigs	4/3.41 (3; 4)	3/2.83 (2, 4)^***^
I could adopt my pig farming in order to have pigs ready for sale at specific times of the year	3/2.89 (2; 4)	2/2.63 (2; 3)^***^
It is possible for me to tell visitors not to enter in the pig-house with their own boots	4/3.60 (3; 4)	4/3.77 (3; 5)^*^
Attitude		
I have lost confidence in pig production	1/1.51 (1; 2)	1/1.35 (1; 1)^***^
I feel more optimistic about the pig enterprise	5/4.38 (4; 5)	5/4.48 (4; 5)^*^
Other		
There has been an increase in disputes, disagreements or jealousy among my neighbours	1/1.79 (1; 2)	1/1.73 (1; 2)

^*^ = *p* ≤ 0.1; ^**^ = *p* < 0.05; ^***^ = *p* < 0.01

^a^Perceptions were measured as agreements with given statements according to a Likert-scale with five levels: strongly agree = 5; agree = 4; neither agree nor disagree = 3; Disagree = 2; Strongly disagree = 1. Changes in perception between the two interview occasions were evaluated using the paired Wilcoxon signed-rank Test

^b^Median/mean (1st; 3rd quarantile)

^c^*ASF* African swine fever

#### Change in perceptions over time

**Fig. 1:**
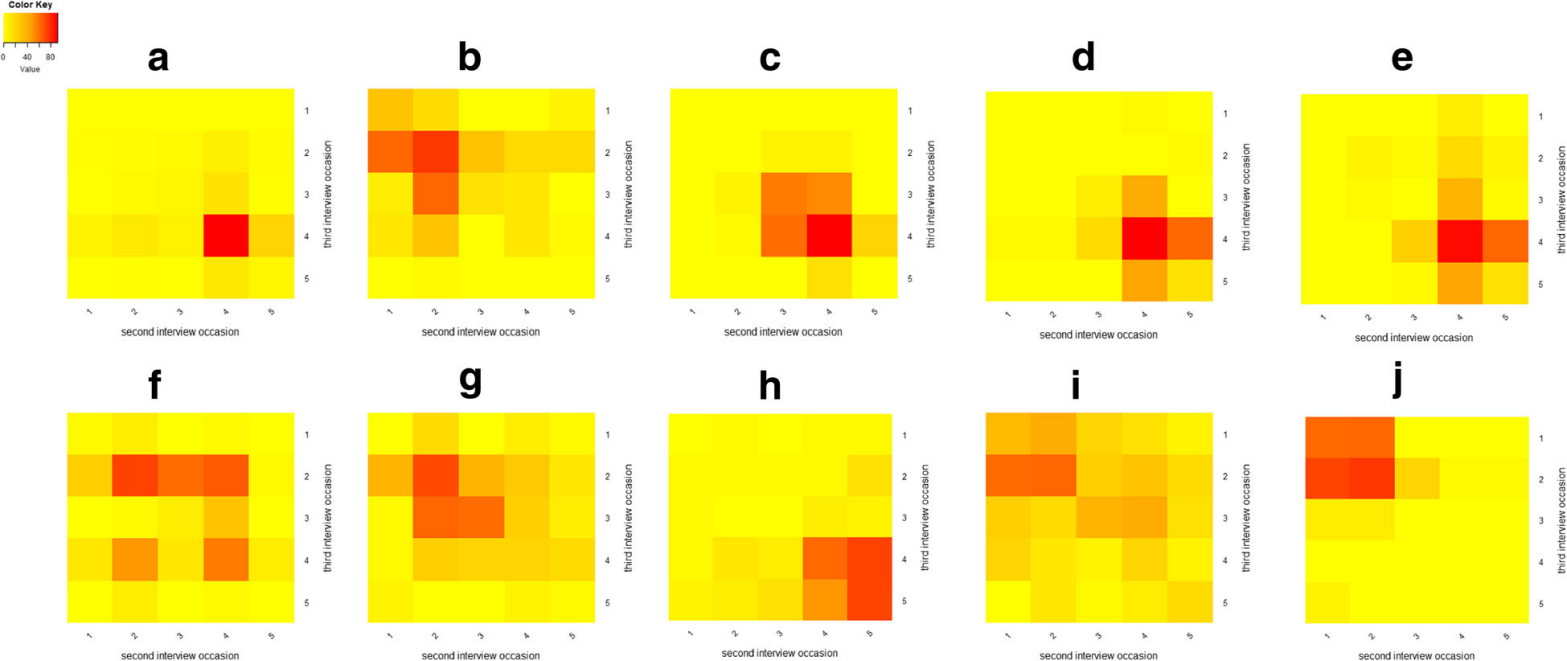
Heatmaps indicating the number of respondents (*n* = 196) for each level of agreement to 10 statements (**a**-**j**), on two interview occasions in a longitudinal interview study conducted on household level in northern Uganda between 2014 and 2015. The level of agreement with the given statements was recorded on a Likert-scale with five levels: strongly agree = 5; agree = 4; neither agree nor disagree = 3; Disagree = 2; Strongly disagree = 1. The statements were: **a** I think it is possible to protect my pigs from getting ASF by improving farm biosecurity. **b** ASF can not be prevented. **c** I would like to invest in farm biosecurity if I received advice on what to do. **d** Improved farm biosecurity improves pig health and pig growth. **e** Buying live pigs is a risky behaviour for contracting ASF. **f** Frequent selling and buying of pigs is necessary for successful pig farming. **g** Eating pork from pigs that have died from ASF is safe for human health. **h** I don’t want to eat or buy pork from pigs that have died from ASF. **i** Cooking kills the ASF virus. **j** If pork prices are lower in the neighbouring village due to them having an outbreak of ASF I will buy my pork there

For the ten statements on general biosecurity, between 56 and 130 respondents changed their answers between the two interview occasions, and similarly for the other categories of statements. For some statements (two out of ten statements on general biosecurity, two out of five on control interventions and one statements on attitudes) a significant change in the observed level of agreement occurred between the two interview occasions, see Table 1 and Fig. 1. Although significant, the mean differences were generally small.

### Associations between perceptions on biosecurity and actions

No significant associations were identified between the perceptions on biosecurity, expressed as level of agreement with the statements, and actions related to biosecurity, see Table 2.

**Table 2:** Associations between perceptions on general biosecurity and related pig farming actions critical for biosecurity

Biosecurity statement/action			I think it is possible to protect my pigs from getting ASF by improving biosecurity^a, d^		ASF can not be prevented^a^		I would like to invest in biosecurity if I received advice^a^		Improved biosecurity improves pig health and growth^a^		Buying live pigs is a risk behaviour for contracting ASF^a^		Frequent selling and buying of pigs is necessary for successful pig farming^a^	
	Second interview occasion^b^	Third interview occasion^b^	Second interview occasion^c^	Third interview occasion^c^	Second interview occasion^c^	Third interview occasion^c^	Second interview occasion^c^	Third interview occasion^c^	Second interview occasion^c^	Third interview occasion^c^	Second interview occasion^c^	Third interview occasion^c^	Second interview occasion^c^	Third interview occasion^c^
HH^d^ had expenditure for biosecurity equipment	22	13	4/3.55*	4/3.78	2/2.41	2/2.30	4/3.73	4/3.77	4/3.86	4/4.00	4/3.77	4/3.92	2/2.55	3/3.08**
HH had no expenditure for biosecurity equipment	173	181	4/3.83	4/3.80	2/2.33	2/2.13	4/3.80	4/3.81	4/4.01	4/4.09	4/3.88	4/4.11	2/2.58	2.5/2.83
HH Bought pigs	48	38	4/3.92**	4/3.79	2/2.46	2/2.00	4/3.75	4/3.73	4/3.94	4/4.05	4/3.81	4/4.03	2/2.54	3/2.97
HH did not buy pigs	148	158	4/3.78	4/3.79	2/2.30	2/2.19	4/3.80	4/3.82	4/4.00	4/4.10	4/3.89	4/4.12	2/2.59	3/2.81
HH Sold pigs	127	92	4/3.82	4/3.92	2/2.43	2/1.97	4/3.78	4/3.74	4/3.95	4/4.01	4/3.85	4/4.01	2/2.63	2/2.78
HH did not sell any pigs	62	101	4/3.71	4/3.67	2/2.21	2/2.31	4/3.77	4/3.85	4/4.07	4/4.14	4/3.87	4/4.14	2/2.48	3/2.88

^*^ = *p* < 0.1; ^**^ = *p* < 0.05

^a^Perceptions were measured as agreements with given statements according to a Likert-scale with five levels: strongly agree = 5; agree = 4; neither agree nor disagree = 3; Disagree = 2; Strongly disagree = 1. Associations between perceptions and actions were evaluated using the Kruskal Wallis test

^b^Number of respondents

^c^Median/mean

^d^*ASF* African swine fever, *HH* household

### Associations between perceptions on general biosecurity, control interventions and attitudes, and socio-economic factors and outbreaks of ASF

No significant association between the perceptions and selected socio-economic factors were found. A significant negative association was found between the statement “I don’t want to eat or buy pork from pigs that have died from ASF” and ASF outbreaks on the third interview occasion, see Additional file 4. Trends in associations consistent for the two interview occasions were found for the statements “I think it is possible to protect my pigs from getting ASF by improving biosecurity” and “I would be happy to buy pork products from a slaughterhouse that receives pigs that have been in contact with pigs dying from ASF”, see Table 3. The level of agreement with “I think it is possible to protect my pigs from getting ASF by improving biosecurity” was negatively associated with outbreaks of ASF whereas the level of agreement with “I would be happy to buy pork products from a slaughterhouse that receives pigs that have been in contact with pigs dying from ASF” was positively associated with outbreaks of ASF.

**Table 3:** Associations between perceptions and outbreaks of ASF

Statement^a^	Perception^b^	
	Second occasion^c^	Third Occasion^c^
General biosecurity		
I think it is possible to protect my pigs from getting ASF^d^ by improving farm bio security		
ASF outbreak second interview occasion	4/3.40^**^	4/3.73
No ASF outbreak second interview occasion	4/3.83	4/3.79
ASF outbreak third interview occasion	NA	4/3.43^**^
No ASF outbreak third interview occasion	NA	4/3.84
Control interventions		
I would be happy to buy pork products from a slaughterhouse that receive pigs that have been in contact with pigs dying from ASF		
ASF outbreak second interview occasion	2/2.13^*^	2/1.73
No ASF outbreak second interview occasion	2/1.71	2/1.66
ASF outbreak third interview occasion	NA	2/2.00^*^
No ASF outbreak third interview occasion	NA	2, 1.62

^*^ = *p* = 0.1; ^**^ = *p* < 0.05

^a^Associations consistent over the two interview occasions are shown

^b^Perceptions were measured as agreements with given statements according to a Likert-scale with five levels: strongly agree = 5; agree = 4; neither agree nor disagree = 3; Disagree = 2; Strongly disagree = 1. Associations were evaluated using the Kruskal Wallis test

^c^Median/mean

^d^*ASF* African swine fever

#### Associations between change in perception and ASF outbreaks

The associations between change in perception and ASF outbreaks were calculated for the five statements with significant changes over time (*p* < 0.01), as well as for those with *p*-values indicating such a change (*p* < 0.1), see Tables 1 and 4. A significant negative association was found between the change in agreement for the biosecurity related statement “I don’t want to eat or buy pork from pigs that have died from ASF” and households that had suffered ASF outbreaks between the second and third interview. The change in level of agreement with the statement concerning attitude to pig production “I feel more optimistic about the pig enterprise” was significantly positively associated with ASF outbreak between the first and second interview. This significant association was confirmed by the statement formulated in the opposite direction.

**Table 4:** Associations between change in perceptions between the second and third interview occasion, and ASF outbreaks

Statement^a^	Change in perception between interview occasions^b, c^
General biosecurity	
ASF^d^ can not be prevented	o/− 0.179
ASF outbreak second interview occasion	0/− 0.200
No ASF outbreak second interview occasion	-1/− 0.178
ASF outbreak third interview occasion	0/− 0.217
No ASF outbreak third interview occasion	−0.5/− 0.174
Improved farm biosecurity improves pig health and pig growth	0/0.098
ASF outbreak second interview occasion	0/−0.133
No ASF outbreak second interview occasion	0/0.118
ASF outbreak third interview occasion	0/0.217
No ASF outbreak third interview occasion	0/0.082
Buying live pigs is a risk behaviour for contracting ASF	0/0.226
ASF outbreak second interview occasion	0/0.400
No ASF outbreak second interview occasion	0/0.211
ASF outbreak third interview occasion	0/0.435
No ASF outbreak third interview occasion	0/0.198
Frequent selling and buying of pigs is necessary for successful pig farming	0/0.226
ASF outbreak second interview occasion	0/0.400
No ASF outbreak second interview occasion	0/0.211
ASF outbreak third interview occasion	0/0.435
No ASF outbreak third interview occasion	0/0.198
I dont want to eat or buy pork from pigs that have died from ASF	0/0.167
ASF outbreak second interview occasion	1/0.667^*^
No ASF outbreak second interview occasion	0/0.124
ASF outbreak third interview occasion	0/−0.870^***^
No ASF outbreak third interview occasion	0/0.308
If pork prices are lower in the neighbouring village due to them having an outbreak of ASF I will buy my pork there	0/−0.190
ASF outbreak second interview occasion	0/−0.267
No ASF outbreak second interview occasion	0/−0.183
ASF outbreak third interview occasion	0/0.000
No ASF outbreak third interview occasion	0/−0.215
Control interventions	
If I would get a fair price I would be willing to sell all my healthy pigs when an ASF outbreak occurred in the area	0/−0.133
ASF outbreak second interview occasion	0/0.133
No ASF outbreak second interview occasion	0/−0.156
ASF outbreak third interview occasion	0/0.000
No ASF outbreak third interview occasion	0/−0.151
I can choose where to/to whom I sell my pigs	0/−0.583
ASF outbreak second interview occasion	−1/−0.600
No ASF outbreak second interview occasion	0/−0.582
ASF outbreak third interview occasion	−0.5/− 0.727
No ASF outbreak third interview occasion	0/−0.565
I could adopt my pig farming in order to have pigs ready for sale at specific times of the year	0/−0.259
ASF outbreak second interview occasion	0/−0.133
No ASF outbreak second interview occasion	0/−0.270
ASF outbreak third interview occasion	0/−0.391
No ASF outbreak third interview occasion	0/−0.241
It is possible for me to tell visitors not to enter in the pig-house with their own boots	0/0.176
ASF outbreak second interview occasion	1/0.733^*^
No ASF outbreak second interview occasion	0/0.129
ASF outbreak third interview occasion	0/0.091
No ASF outbreak third interview occasion	0/0.187
Attitudes	
I have lost confidence in pig production	0/−0.161
ASF outbreak second interview occasion	0/−0.600^**^
No ASF outbreak second interview occasion	0/−0.124
ASF outbreak third interview occasion	0/0.130^**^
No ASF outbreak third interview occasion	0/−0.200
I feel more optimistic about the pig enterprise	0/0.103
ASF outbreak second interview occasion	0/0.667^***^
No ASF outbreak second interview occasion	0/0.056
ASF outbreak third interview occasion	0/0.000
No ASF outbreak third interview occasion	0/0.117

^*^ = *p* = 0.1; ^**^ = *p* < 0.05; ^***^ = *p* < 0.01

^a^Statements with *p*-values below 0.1 for the change in perception between the second and third interview occasion are displayed

^b^Perceptions were measured as agreements with given statements according to a Likert-scale with five levels: strongly agree = 5; agree = 4; neither agree nor disagree = 3; Disagree = 2; Strongly disagree = 1. Associations between the magnitude of the change and outbreaks of African swine fever were evaluated using the Kruskal Wallis test

^c^Median/mean

^d^*ASF* African swine fever

## Discussion

The situation in the study area is challenging in several ways, including elements such as post-conflict vulnerability [24], deep poverty [6, 25] and endemic ASF-infections [7]. Despite this, the respondents’ answers generally convey positivism and hope. This applies to statements regarding the future prospects of pig farming as well as the potential of biosecurity for preventing outbreaks of ASF. Outbreaks of ASF, however, seemed to reduce the optimism about the preventive potential of farm biosecurity. On the third interview occasion this was expressed as higher level of agreement with the statement expressing lost hope in pig farming, lower trust in the possibility to use biosecurity to protect against ASF, and lower level of willingness to invest in biosecurity, among households that had suffered ASF outbreaks. Similar observations were made after the outbreak of Foot and mouth disease in the United Kingdom (UK) in 2001 [26]. In that study, respondents expressed lost courage and general feelings of hopelessness after the outbreak. As in our study, however, the UK respondents also managed to see some positive side effects of the outbreak, exemplified as structural changes and modernization already being underway happening faster because of the outbreak. In a previous study including the same households as the one reported here, we observed short-term positive economic impacts of ASF outbreaks, as well as a tendency towards higher pork consumption in households with recent ASF outbreaks [27]. Previous studies also confirm that both in-contact and diseased pigs are traded and slaughtered in outbreak situations, and pork from diseased or dead pigs is consumed, bartered or sold [19, 27, 28]. Contrasting this, statements regarding the households’ willingness to, and perceived safety of, consuming pork from ASF infected pigs confirmed that respondents generally do not want to consume such pork, and that they think that ASF has a zoonotic potential and that the virus is not inactivated by cooking. However, households that had experienced outbreaks on the third interview occasion, and thus probably had consumed pork from pigs that were infected or dying from ASF, were less negative towards consumption, and agreed more with the statement on the non-zoonotic potential of ASF. These perceptions on public health in connection with ASF, some of them false, are probably contributing to the generally low acceptance for the hypothetical control interventions reflected in the statements. In addition to being unwilling to buy heat-treated, and thus safe, pork from operations that have slaughtered ASF-in-contact-pigs, respondents did not want to sell their heathy pigs to such operations, even if an outbreak was present in the area and pig prices were fair. Households that had experienced outbreaks on the third interview occasion, and thus had been exposed to the impact of outbreaks, were however more willing to sell their healthy pigs. In other communities, and for other animal species, it is well known that the number of animals carries an important social role [29]. This might have contributed to the reluctance to sell healthy animals, as might a generally optimistic attitude, expressed by one respondent as “*I don’t want to sell my healthy pigs because they might not fall sick after all*”. Other control interventions, such as choosing the trade partner, and being able to have pigs ready for slaughter at specific times of the year seemed to be associated with educational level of the spouse and poverty, respectively. This probably illustrates the complex livelihood situations of the study population [30], where decisions regarding pig farming and disease control are governed by multiple factors, all of which we did not aim to investigate in the current study. In a study on anthrax from western Uganda it was shown how decision making in connection with disease control was mainly affected by factors other than knowledge about the disease, laws and regulations [31]. Poverty is clearly a relevant factor in this regard, but in the current study the poverty score was not significantly associated with any of the investigated perceptions. If the control interventions reflected in the included statements are to be promoted, information, communication and their adaptation to local situations are needed, as our results indicate that they are not highly accepted by the population as presented.

The ability of people, without formal schooling, to describe, recognize and control diseases of importance for them is no longer questioned [32–34]. This has also been proven for the study population and ASF in particular [19]. However, knowledge gaps still exist. Addressing these, for example regarding the zoonotic potential of ASF, and the ability of the virus to survive cooking, will be an important part of increasing acceptance and enhancing implementation of the hypothetical control interventions reflected in the included statements.

The authors argue that safe slaughter is key among these control interventions. Currently, pig slaughter in the study area happens mostly without veterinary inspection at village slaughter slabs without possibility for almost any biosecurity. Offal are often let on the ground, in exposure to free roaming pigs or dogs. ASF infected pigs will be slaughtered, thus it is of paramount importance that slaughter can be done without transmitting the disease further, i.e. with minimal blood contamination of the surroundings, and proper management of offal and the pork products. If slaughter could be done in a biosecure way, and the pork transferred into safe products for which there was a market, diseased pigs could be bought for slaughter at decent prices, and the operation made sustainable. This way of allowing trade based on the individual risk of the commodities and the local situation, and not according to guidelines set up for international trade agreements where acceptable risk levels differ from what is reasonable in endemic situations, have been proposed as part of a sustainable development solution for poor countries [35–37].

As outbreaks in the study area are seasonal with yearly peaks at Easter, Independence Day (1st October) and Christmas (also coinciding with the dry period when pigs are let free to roam for food) [19], adaptation of the pig rearing to have pigs ready for slaughter before expected outbreaks, could be an effective way to avoid outbreaks for the individual farmer. Acceptance for the statement reflecting this control intervention was moderate. Planned pig rearing, however, require access to, and ability to purchase, feed. In the previous study including the same smallholders, average yearly spending on pig feed were 19 USD, and feed availability frequently mentioned as a challenge [27]. Even if the statement reflecting an obligatory change of footwear before accessing the pigs was highly accepted, this control intervention will not be an effective in a pig population that mostly are not stabled.

We observed a high inconsistency of perceptions between the two interview occasions. However, these changes were mostly not associated with households’ experience of ASF outbreaks. The change in perception towards eating pork from ASF infected pigs, however, was associated with outbreak experience. In this regard recent and more distant outbreaks seem to have opposite impacts on the perception, perhaps reflecting a recall bias [38], or a reversion to the socially desired response [39] as memories of the outbreak effects fade away. This difference between past and recent outbreak effects was also seen for statements reflecting the optimism in pig production. Again, the more distant outbreaks did not seem to have created a prolonged negative impression, whereas the more recent ones still seemed to carry negative impression.

No significant associations were found between the three selected actions that were relevant for farm biosecurity, and related statements about biosecurity. Paradoxically, on the second interview occasion higher agreement with the statement on the preventive potential of biosecurity seemed to be associated with not having expenditure for biosecurity. It should be noted that the distribution of responses to this question was skewed, and that the average expenditure for biosecurity was very small, for most households representing only of a pair of rubber boots [27]. As most pigs in the study area roam free at least some part of the year [19], the potential of this expenditure to prevent ASF will be limited, and the respondents might not even have regarded the purchase of rubber boots as a biosecurity expenditure although it was coded as such in this study. On the same interview occasion, respondents that had bought pigs seemed to agree to a higher degree with the statement on the preventive potential of biosecurity compared to respondents who had not bought pigs. This might reflect that the respondents know that trade is a risky activity, and put their trust in biosecurity as a protective measure. Similarly, on the third interview occasion, respondents that had expenditure for biosecurity seemed to agree to a higher degree with the statement regarding the necessity of frequent trade for success in pig farming.

Methodologically some remarks should be made. No statistical testing was carried out for consistency and reliability of the questions, potentially limiting the validity of the results. These aspects were instead assessed by studying questions with control statements formulated in the opposite way. However, the first author observed difficulties for both facilitators and respondents in grasping questions involving agreement to a negative statement, such as “ASF cannot be prevented”. Hence, the results for such statements have to be thoughtfully considered. However, the comparison with the control statements, formulated in the opposite way, showed expected diverging average results, confirming the validity of the results also for questions that appeared difficult in the observation of the interviews. Bias due to the presence of a socially desirable response [39] must also be considered. This might have been accentuated as the facilitators were local veterinarians and known by some of the respondents, even if the purpose of the study and that it was a research activity and not attached to any governmental disease control activities, was repeatedly underlined. In addition, the effect of the study itself on the results cannot be neglected (although questioned, sometimes called the Hawthorne effect [40]). Finally, to gain more in-depth understanding of people’s behaviour, reasoning and decision-making processes, deeper involvement of the social sciences and a qualitative research approach might have been better suited. However, as a scoping stage, and for enabling the calculations of associations between perceptions and explanatory factors including outbreaks of ASF, the selected method was preferable.

## Conclusions

Outbreaks of ASF reduced smallholders’ positive attitudes towards pig farming, the belief in the potential of biosecurity for preventing outbreaks, and the will to invest in biosecurity. The respondents generally did not want to consume pork from ASF-infected pigs, but were less negative towards consumption after having experienced ASF-outbreaks. Many respondents believed that ASF is zoonotic, and that the virus survive cooking. Communicating correct information in this regard will be important for increasing acceptance and enhancing implementation of the hypothetical control interventions reflected in the statements included in the study. Likewise, participatory development to adopt the control interventions to the local context on community level will be important for successful implementation. To gain in-depth understanding of people’s behaviour, reasoning and decision-making processes, deeper involvement of the social sciences and a qualitative research approach might be used in future research following this study.

## Methods

### Study area

**Fig. 2:**
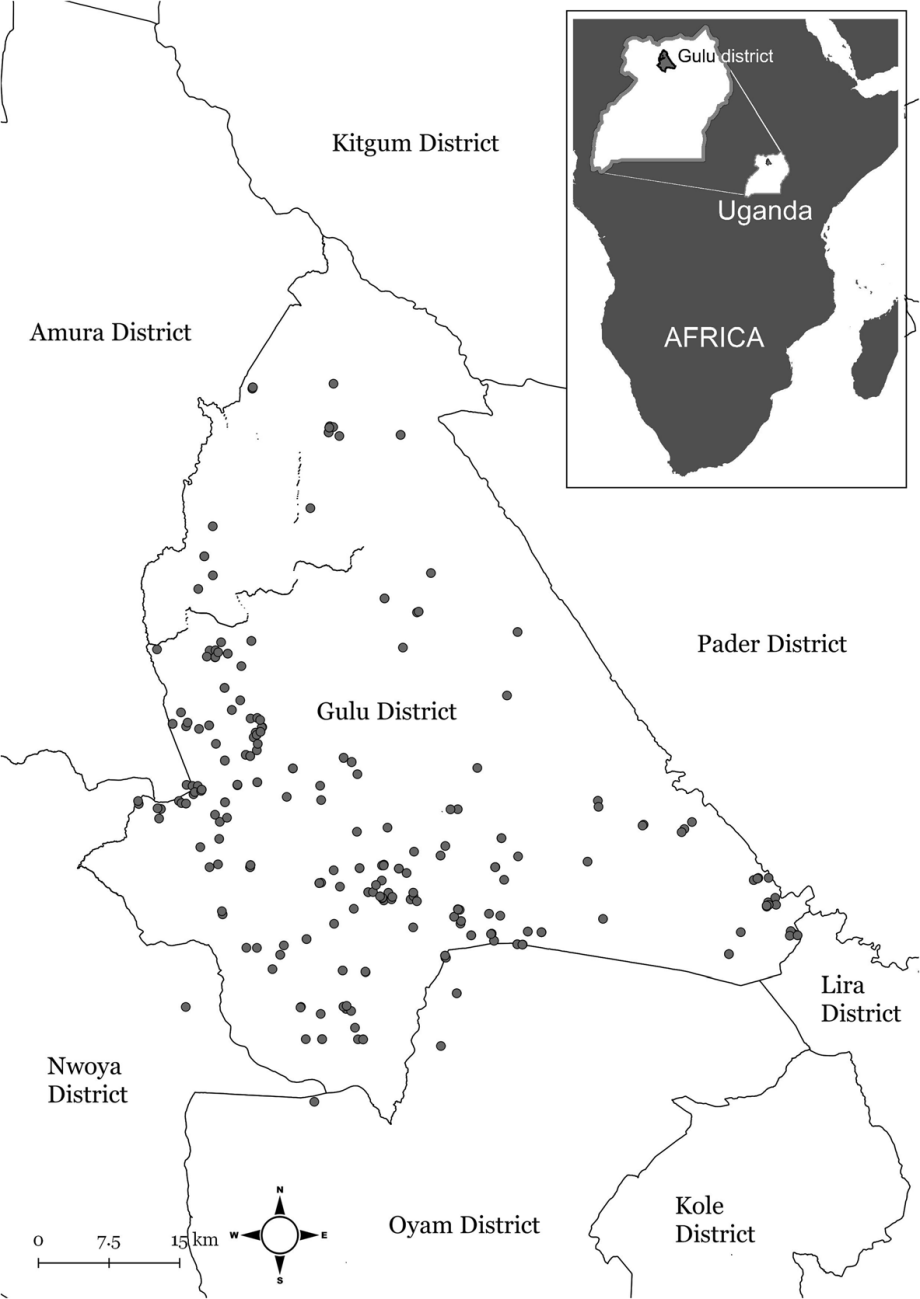
Geographical location of households in a longitudinal interview study conducted on household level in northern Uganda between 2014 and 2015. Included households are marked with grey dots. Figure created by Linda Svensson, National Veterinary Institute, Sweden using GADM, a geographic database of global administrative areas (boundaries) licensed under a Creative Commons Attribution-Noncommercial-Share Alike 3.0 United States License. These files were extracted from GADM version 1.0, in March 2009

The study was implemented in, and close to, Gulu district in northern Uganda (see Fig. 2) from March 2014 to March 2015. At that time Gulu district was administratively divided into two counties, 12 sub-counties, 70 parishes and 294 villages (the smallest administrative unit in Uganda) [41] and covered approximately three and a half thousand km^2^. According to a human population census from 2014 and the equivalent for domestic animals from 2008, the district had approximately 500,000 inhabitants in 90,000 households, including more than 6,000 pig-keeping households [42, 43]. No formal household or animal registry exists in Uganda. According to previous studies, the study population is homogenous in terms of demography, social and economic status, as well as for the pig husbandry system used [27, 44]. The pig production is dominated by an extensive husbandry system with pigs being kept on free range and with an average herd size of less than four pigs [27, 44]. The region has a tropical climate with one rainy season (from April through November) and one dry season (from December to March).

### Study design

In a longitudinal study, structured interviews with 200 randomly selected, pig-keeping households were undertaken three times with a six-month interval. Two types of data were collected. Firstly, on all three interview occasions, data on family and pig herd demographics, social and economic characteristics of the household, pig trade and pig business were collected. These results are presented in a separate article [27]. Secondly, on the second and third interview occasion, data on perceptions relating to general biosecurity, control interventions, attitudes towards pig farming and neighbour relations were collected. Results relating to these data are presented here.

### Participant selection

The participant selection is described in detail previously [27]. Briefly, participant households were randomly selected from a list of pig-owning households previously obtained and including four thousand pig-keeping households covering all 12 subcounties in Gulu district at that time [7]. Only household that could be reached on all three interview occasions were included in the study, replacements were done only at the first interview occasion (selected households being replaced with the closest pig-keeping household in the same village). The respondent was an adult household member who was available at the time, and who had sufficient knowledge of the household’s pig keeping. Whenever possible, the same person in each selected household was interviewed on all three occasions. Interviews were conducted at the respondents’ homes or other nearby places.

### Data collection and handling

Data were collected using structured face-to-face individual interviews noting answers on paper questionnaires. The interviews were conducted in the local language (Luo) by two of the authors (BN and SA), both of whom are native Luo-speakers and proficient in English. Answers were noted in English. The first author of this paper was present at half of the interviews, alternating daily between the two interviewers to provide consistency and supervision. The questionnaires were constructed in English using the software EasyResearch (QuestBack International HQ, Oslo, Norway). Before conducting the interviews, BN and SA came to an agreement on the translation from English to Luo. The questionnaire was pre-tested in two households not included in the study before minor adjustments and final implementation. The focus of the pre-tests was on ensuring that questions were formulated so that the respondents understood them in the intended way, and on removing any non-relevant questions. Data were entered by single entry into a web-based database shortly after each round of interviews (EasyResearch, QuestBack International HQ, Oslo, Norway). A random sample of 10% of the questionnaires was checked for data entry quality by the first author. The questionnaires are displayed as Additional files 1, 2 and 3.

The questions relating to general biosecurity, control interventions, attitudes towards pig farming and neighbour relations were answered as agreements to statements on a Likert scale [45]. The scale had five levels, ranging from “strongly disagree” (=1) to “strongly agree” (=5). Some statements covered the same topic but were phrased in different or opposite ways to check for consistency, e.g. “I think it is possible to protect my pigs from getting ASF by improving biosecurity” versus “ASF cannot be prevented” and “I feel more optimistic about the pig enterprise” versus “I have lost confidence in pig production”. The statements on control interventions were selected based on local knowledge, expert opinion and basic parameters of ASF epidemiology. More specifically the selection was based on the understanding that blood from infected pigs is the most infectious material, and slaughter thus one of the most critical activities for disease transmission [8, 10]. Further, the local setting and characteristics of the pig and trade networks were considered [19, 46–48]. These statements reflected four hypothetical interventions: 1) biosafe slaughter in operations that would buy pigs for slaughter also during outbreak situations, 2) in addition to this, processing of pork into heat treated, and thus ASF-safe, pork products for the local market, 3) adaptation of rearing times to have pigs ready for slaughter before previously identified yearly peaks of outbreaks [19], 4) obligatory change of footwear before accessing the pigs.

### Data analysis

Data were analysed to highlight five different aspects of biosecurity and control interventions according to the objectives of the study:

- Describe perceptions on general biosecurity, control interventions, attitudes on pig business and relation with neighbours
- Assess changes in perceptions over time
- Assess associations between the perceptions and actions critical for biosecurity
- Assess associations between the perceptions, and selected socio-economic factors and outbreaks of ASF
- Assess associations between the changes in perceptions over time and outbreaks of ASF

Change in perceptions between the two interview occasions was calculated as the difference between the two Likert scale scores.

Explanatory factors in the analysis of associations between perceptions, and socio-economic factors and outbreaks of ASF were selected according to results from previous studies [19, 27]. These variables were: household poverty score, number of pigs at the first interview occasion, the gross margin in pig production, the educational level of the household head and of the spouse, and ASF outbreaks occurring between the first and second and second and third interview occasion, respectively. The poverty score, developed by Grameen foundation,^1^ utilises non-financial indicators from Uganda’s national income and expenditures surveys to assess the probability of a household to fall under a selected poverty line [49]. The lower the score, the higher the probability. The index is similar to other asset-based indexes, but also includes for example family size, level of education and land tenure [50]. The gross margin in pig production was calculated as in the previous article by Chenais et al. (2017) based partly on the same data. Briefly it constituted all recorded incomes minus all recorded costs in the pig production. Financial variables were transferred from the local currency (Ugandan shillings, UGX) to USD using the exchange rate for the relevant dates (1 UGX = 0,0003 USD^2^). Education levels of the household head and spouse were recorded separately as four level categorical variables, with the levels no formal education (=1), primary education (=2), secondary education (=3) or a higher level education (=4). The poverty score, the education level and the number of pigs in the household were recorded only at the first interview occasion.

ASF outbreak status (yes/no) was defined for the two time periods covered by the interviews in the present study using the same criteria as in the previous study based partly on the same data [27]. As in that study, the criteria for “yes” was that the respondent described pig deaths and either stated the reason to be ASF, or described clinical signs and epidemiological aspects that unambiguously could attribute the cases to ASF. Uncertain cases were assigned “no” status.

Summary statistics, including measures of central tendency and dispersion, were calculated for all collected variables for the five separate aspects investigated. To avoid drawing false positive conclusions based on type I-error, and as multiple analyses were done, only associations with a *p*-value below 0.01 was considered significant. However, taking into account ASF epidemiology and the mentioned conceptual model, associations with *p*-values below 0.1 were still considered relevant for some analysis [51]. The Kruskal Wallis test was used to assess the associations between the perceptions and selected explanatory variables. Based on the results of these tests, relevant relationships were investigated further for direction and consistency between interview occasions [51]. The paired Wilcoxon signed-rank test was used to assess changes in perceptions between the two interview occasions. The changes in perception were illustrated by heatmaps. The Kruskal Wallis test was used to assess associations between the perceptions and biosecurity actions, between perceptions and explanatory factors, as well as for association between changes in perception and outbreaks of ASF. Data were managed, analysed and visualised using basic packages in R software version 3.1.0 (R Core Team, 2015).

## Additional files

Additional file 1: Questionnaire used in a study conducted with smallholder pig-farmers in northern Uganda 2014–2015. First interview. (DOCX 46 kb)

Additional file 2: Questionnaire used in a study conducted with smallholder pig-farmers in northern Uganda 2014–2015. Second interview. (DOCX 30 kb)

Additional file 3: Questionnaire used in a study conducted with smallholder pig-farmers in northern Uganda 2014–2015. Third interview. (DOC 250 kb)

Additional file 4: Relevant associations between perceptions on general biosecurity, control interventions and attitudes to pig farming, and socio-economic variables and outbreaks of African swine fever (ASF) from a longitudinal interview study conducted with smallholder pig-farmers in northern Uganda 2014–2015. Perceptions were measured as agreements with given statements according to a Likert-scale with five levels: strongly agree = 5; agree = 4; neither agree nor disagree = 3; Disagree = 2; Strongly disagree = 1. Education level of the household head and spouse were recorded as a four-level categorical variable: no formal education =1; primary education =2; secondary education =3; higher level education =4. Associations were evaluated using the Kruskal Wallis test. Levels of significance is marked as * = *p* = 0.1; ** = *p* < 0.05; *** = *p* < 0.01. Associations with *p*-values > 0.1 are not displayed. Continuous explanatory variables are displayed as jittered scatter plots, and as Spearman rank colleration values, for categorical explanatory variables the median and mean value for each category are given. HH = household. (DOCX 91 kb)

## Data Availability

The datasets used and/or analysed during the current study are available from the corresponding author on reasonable request.

## Abbreviations

ASF: African swine fever
HH: Household
UGX: Ugandan shilling
USD: US dollar

## Footnotes

1
http://www.grameenfoundation.org/

2 http://www.xe.com/sv/currencyconverter/convert/?Amount=1&From=UGX&To=USD. Accessed 30th June 2016. Using the exact exchange rate for each interview date did not change the outcome (data not shown), therefore the same exchange rate was used for all dates.

## References

[1] Randolph TF, Schelling E, Grace D, Nicholson CF, Leroy JL, Cole DC, Demment MW, Omore A, Zinsstag J, Ruel M (2007). Invited review: role of livestock in human nutrition and health for poverty reduction in developing countries. J Anim Sci.

[2] Krishna A, Lumonya D, Markiewicz M, Mugumya F, Kafuko A, Wegoye J (2006). Escaping poverty and becoming poor in 36 villages of central and Western Uganda. J Dev Stud.

[3] Herrero M, Grace D, Njuki J, Johnson N, Enahoro D, Silvestri S, Rufino MC (2013). The roles of livestock in developing countries. Animal.

[4] Mehta-Bhatt P, FicMehta-Bhatt P, Ficarelli PP, Herring R (2014). Livestock in the food debate. Oxford Handbook of Food, Politics, and Society. Volume 1–13, edn.

[5] Penrith ML, Vosloo W, Jori F, Bastos AD (2013). African swine fever virus eradication in Africa. Virus Res.

[6] Chenais E (2017). African swine fever in Uganda: epidemiology and socio-economic impact in the smallholder setting. Dissertation summary.

[7] Chenais E, Sternberg-Lewerin S, Boqvist S, Emanuelson U, Aliro T, Tejler E, Cocca G, Masembe C, Ståhl K. African swine fever in Uganda: qualitative evaluation of three surveillance methods with implications for other resource-poor settings. Front Vet Sci. 2015;2. 10.3389/fvets.2015.00051. 10.3389/fvets.2015.00051 PMC4673915 26664978

[8] Costard S, Wieland B, de Glanville W, Jori F, Rowlands R, Vosloo W, Roger F, Pfeiffer DU, Dixon LK (2009). African swine fever: how can global spread be prevented?. Philos Trans R Soc Lond Ser B Biol Sci.

[9] Greig A, Plowright W (1970). The excretion of two virulent strains of African swine fever virus by domestic pigs. J Hyg.

[10] Guinat C, Gogin A, Blome S, Keil G, Pollin R, Pfeiffer DU, Dixon L (2016). Transmission routes of African swine fever virus to domestic pigs: current knowledge and future research directions. Vet Rec.

[11] Montgomery E (1921). On a form of swine fever occuring in British East Africa (Kenya colony). J Comp Pathol Ther.

[12] Steyn D (1932). East African virus disease in pigs. Rep Dir Vet Serv Animal Ind Union S Afr.

[13] McKercher PD, Yedloutschnig RJ, Callis JJ, Murphy R, Panina GF, Civardi A, Bugnetti M, Foni E, Laddomada A, Scarano C (1987). Survival of viruses in “prosciutto di Parma” (Parma ham). Can Institute Food Sci Technol J.

[14] Mebus CA, Arias M, Pineda JM, Tapiador J, House C, Sanchez-Vizcaino F (1997). Survival of several porcine viruses in different Spanish dry-cured meat products. Food Chem.

[15] Takamatsu H, Martins C, Escribano J, Alonso C, Dixon L, Salas M, Revilla Y, King A, Adams M, Carstens E, Lefkowitz E (2012). Asfaviridae. Virus taxonomy: Ninth Report of teh International Commitee on Taxonomy of Viruses. edn.

[16] Penrith ML, Vosloo W (2009). Review of African swine fever: transmission, spread and control. J S Afr Vet Assoc.

[17] Barongo MB, Stahl K, Bett B, Bishop RP, Fevre EM, Aliro T, Okoth E, Masembe C, Knobel D, Ssematimba A (2015). Estimating the basic reproductive number (R0) for African swine fever virus (ASFV) transmission between pig herds in Uganda. PLoS One.

[18] Korennoy FI, Gulenkin VM, Gogin AE, Vergne T, Karaulov AK (2016). Estimating the basic reproductive number for African swine fever using the Ukrainian historical epidemic of 1977. Transbound Emerg Dis.

[19] Chenais E, Boqvist S, Sternberg-Lewerin S, Emanuelson U, Ouma E, Dione M, Aliro T, Crafoord F, Masembe C, Stahl K (2017). Knowledge, attitudes and practices related to African swine fever within smallholder pig production in northern Uganda. Transbound Emerg Dis.

[20] Chenais E, Sternberg-Lewerin S, Boqvist S, Liu L, LeBlanc N, Aliro T, Masembe C, Ståhl K (2017). African swine fever outbreak on a medium-sized farm in Uganda: biosecurity breaches and within-farm virus contamination. Trop Anim Health Prod.

[21] Nantima N, Davies J, Dione M, Ocaido M, Okoth E, Mugisha A, Bishop R (2016). Enhancing knowledge and awareness of biosecurity practices for control of African swine fever among smallholder pig farmers in four districts along the Kenya-Uganda border. Trop Anim Health Prod.

[22] Chambers Robert (2009). Going to Scale with Community-Led Total Sanitation: Reflections on Experience, Issues and Ways Forward. IDS Practice Papers.

[23] Tumwebaze IK, Mosler HJ (2015). Effectiveness of group discussions and commitment in improving cleaning behaviour of shared sanitation users in Kampala, Uganda slums. Soc Sci Med.

[24] Finnström S (2008). Living with bad surroundings. War, History, and Everyday Moments in Northern Uganda.

[25] Okidi J, McKay A (2003). Poverty dynamics in Uganda: 1992 to 2000.

[26] Scott A, Christie M, Midmore P (2004). Impact of the 2001 foot-and-mouth disease outbreak in Britain: implications for rural studies. J Rural Stud.

[27] Chenais E, Boqvist S, Emanuelson U, von Bromssen C, Ouma E, Aliro T, Masembe C, Stahl K, Sternberg-Lewerin S (2017). Quantitative assessment of social and economic impact of African swine fever outbreaks in northern Uganda. Prev Vet Med.

[28] Dione M, Ouma E, Opio F, Kawuma B, Pezo D (2016). Qualitative analysis of the risks and practices associated with the spread of African swine fever within the smallholder pig value chains in Uganda. Prev Vet Med.

[29] Kaufmann B, Lelea M, Hulsebusch C (2016). Diversity in livestock resources in pastoral systems in Africa. Rev Sci Tech.

[30] Chenais E, Fischer K. Increasing the Local Relevance of Epidemiological Research: Situated Knowledge of Cattle Disease Among Basongora Pastoralists in Uganda. Front Vet Sci. 2018;5(119). 10.3389/fvets.2018.00119 PMC6008553 29951490

[31] Coffin JL, Monje F, Asiimwe-Karimu G, Amuguni HJ, Odoch T (2015). A one health, participatory epidemiology assessment of anthrax (bacillus anthracis) management in Western Uganda. Soc Sci Med.

[32] Mariner JC, Roeder PL (2003). Use of participatory epidemiology in studies of the persistence of lineage 2 rinderpest virus in East Africa. Vet Rec.

[33] Grace D, Randolph T, Affognon H, Dramane D, Diall O, Clausen PH (2009). Characterisation and validation of farmers’ knowledge and practice of cattle trypanosomosis management in the cotton zone of West Africa. Acta Trop.

[34] Catley A, Chibunda RT, Ranga E, Makungu S, Magayane FT, Magoma G, Madege MJ, Vosloo W (2004). Participatory diagnosis of a heat-intolerance syndrome in cattle in Tanzania and association with foot-and-mouth disease. Prev Vet Med.

[35] Naziri D, Rich KM, Bennett B (2015). Would a commodity-based trade approach improve market access for Africa? A case study of the potential of beef exports from communal areas of Namibia. Dev Policy Rev.

[36] Thomson GR, Penrith ML, Atkinson MW, Thalwitzer S, Mancuso A, Atkinson SJ, Osofsky SA (2013). International trade standards for commodities and products derived from animals: the need for a system that integrates food safety and animal disease risk management. Transbound Emerg Dis.

[37] Thomson GR, Tambi EN, Hargreaves SK, Leyland TJ, Catley AP, van ‘t Klooster GG, Penrith ML (2004). International trade in livestock and livestock products: the need for a commodity-based approach. Vet Rec.

[38] Coughlin SS (1990). Recall bias in epidemiologic studies. J Clin Epidemiol.

[39] Paulhus DL, Robinson JP, Shaver PR, Wrightsman LS (1991). Measurement and Control of Response Bias. Measures of Personality and Social Psychological Attitudes.

[40] Wickström G, Bendix T (2000). The “Hawthorne effect”—what did the original Hawthorne studies actually show?. Scand J Work Environ Health.

[41] Gulu district local government statistical abstract 2012/13 [http://www.google.se/url?sa=t&rct=j&q=&esrc=s&source=web&cd=3&ved=0CDMQFjAC&url=http%3A%2F%2Fwww.ubos.org%2Fonlinefiles%2Fuploads%2Fubos%2F2009_HLG_%2520Abstract_printed%2FCIS%2BUPLOADS%2FHigher%2520Local%2520Government%2520Statistical%2520Abstracts_2014%2FGulu.pdf&ei=ybvQVIu_EeS_ygOWi4LoDQ&usg=AFQjCNHUncuPoNAsNQxVomKcy4evzGfKHA&bvm=bv.85076809,d.bGQ]. Accessed 3 Feb 2015.

[42] UBOS (2008). The national livestock census report 2008. Ministry of Agriculture, Animal Industry & Fisheries, Entebbe, Uganda and Uganda Bureau of Statistics, Kampala, Uganda.

[43] UBOS: National population and housing census 2014. Provisonal results [https://www.ubos.org/onlinefiles/uploads/ubos/NPHC/NPHC%202014%20PROVISIONAL%20RESULTS%20REPORT.pdf]. Accessed 19 Mar 2015.

[44] Ikwap K, Jacobson M, LUndeheim N, Owiny DO, Nasinyama GW, Fellström C, Erume J (2014). Characterization of pig production in Gulu and Soroti districts in northern and eastern Uganda. Livest Res Rural Dev.

[45] Likert R (1932). A technique for the measurement of attitudes. Arch Psychol.

[46] Costard S, Zagmutt F, Porphyre T, Roger F, Pfeiffer DU (2012). Small-scale pig farmers’ behaviour, silent release of African swine fever and consequences for persistence. International Symposia on Veterinary Epidemiology and Economics 2012.

[47] Ouma E, Dione M, Lule P, Rosel K, Pezo D. Characterization of smallholder pig production systems in Uganda: constraints and opportunities for engaging with market systems. Livest Res Rural Dev. 2014;26(3). No. 309-2016-525.

[48] Dione M, Oumaa E (2014). K R, J K, P L, D P. participatory assessment of animal health and husbandrypractices in smallholder pig production systems in three highpoverty districts in Uganda. Prev Vet Med.

[49] Progress out of poverty [http://www.progressoutofpoverty.org/]. Accessed 19 Nov 2014.

[50] Hjelm L, Mathiassen A, Wadhwa A. Measuring Poverty for Food Security Analysis: Consumption- Versus Asset-Based Approaches. Food Nutr Bull. 2016:1–15 [Epub ahead of print]. 10.1177/0379572116653509 27334773

[51] Amrhein V, Greenland S, McShane B (2019). Scientists rise up against statistical significance. Nature.

