# Supplementary material for: Smallholders’ perceptions on biosecurity and disease control in relation to African swine fever in an endemically infected area in Northern Uganda
Source: BMC Vet Res. 2019 Aug 5;15:279. doi: 10.1186/s12917-019-2005-7 (PMC6683333; doi:10.1186/s12917-019-2005-7)
Supplement: Supplementary file 1 — Questionnaire used in a study conducted with smallholder pig-farmers in northern Uganda 2014–2015. First interview. (DOCX 46 kb) [file 12917_2019_2005_MOESM1_ESM.docx]

# SLU-SVA-ILRI Socio-economical impacts of African swine fever

Household study, first visit

## Identification

| 1.1. Questionnaire ID |  | | |
| --- | --- | --- | --- |
| 1.2. Date of Survey (DD/MM/YYYY) |  | | |
| 1.3. Enumerator Name |  | | |
| 1.4. Time interview started (HH:MM) |  | | |
| 1.5. Time interview ended (HH:MM) |  | | |
| 1.6. Name of the head of the household |  | | |
| 1.7. Respondent’s name |  | | |
| 1.8. Respondent’s telephon number |  | |  |
| 1.9. Gender of respondent |  | | 1=Male, 2=Female |
| 1.10. Marital status of household head |  | | 1=Married  2=Widow/Widower  3=Single parent  4=Other __________________ |
| 1.11. Name of sub-county |  | | |
| 1.12. Name of parish |  | | |
| 1.13. Name of village |  | | |
| 1.14. GPS Coordinates | Latitude (N/S): | Longitude (E/W): | |

## Household details

2.1. Details of household members (including the household head). **[***WE DEFINE A “HOUSEHOLD” TO INCLUDE ALL MEMBERS OF A COMMON DECISION MAKING UNIT (USUALLY WITHIN ONE RESIDENCE) THAT ARE SHARING INCOME AND OTHER RESOURCES*

|  | **Members of your household**  [*FIRST NAMES]* | | **Year of birth** | **Gender**  (1=Male 2=Female) | **Relationship to household head** | **Highest education level attained** | | **Primary source of income** |
| --- | --- | --- | --- | --- | --- | --- | --- | --- |
| 1 |  | |  |  |  |  | |  |
| 2 |  | |  |  |  |  | |  |
| 3 |  | |  |  |  |  | |  |
| 4 |  | |  |  |  |  | |  |
| 5 |  | |  |  |  |  | |  |
| 6 |  | |  |  |  |  | |  |
| 7 |  | |  |  |  |  | |  |
| 8 |  | |  |  |  |  | |  |
| 9 |  | |  |  |  |  | |  |
| 10 |  | |  |  |  |  | |  |
| 11 |  | |  |  |  |  | |  |
| 12 |  | |  |  |  |  | |  |
| 13 |  | |  |  |  |  | |  |
| **Relationship to household head**  1 = Head  2 = Spouse  3 = Child  4 = Sibling  5 = Parent  6 = Grandchild  7 = Other relative  8 = Non-relative (including employees who live in house)  9 = Other,  specify__________________ | | **Highest education level attained**  0 = No formal education  1 = Nursery  2 = Pre-school age  3 = Primary education (P1-P4)  4 = Primary education (P5-P7)  5 = Secondary school (S1-S2)  6 = Secondary school (S3-S4)  7 = High school (S5-S6)  8 = Vocational training (specify no of years) _________  9 = Tertiary training specify yrs _________  10 = University degree (undergraduate)  11 = University degree (postgraduate)  12=Adult literacy  13=Other, specify ________________________ | | | | | **Primary source of income**  0 = None  1 = Crop farming  2 = Pig keeping (incl. sales)  3 = Cattle keeping  4 = Poultry/keeping (inc. sales)  5 = Salaried employment  6 = Self-employed-off farm  7 = Casual laborer  8 = Boda boda  9 = Student/pupil  10 = Charcoal burning  11 = Pre-school age  12 = Other, specify_______________ | |

2.2. Further details for children of school age:

| **Name**  (copy from above) | **Type of school**  (code) | **Cost per term** | **Number of missed schooldays during last term** | **Reason for**  **missed**  **schooldays**  (code) |
| --- | --- | --- | --- | --- |
|  |  |  |  |  |
|  |  |  |  |  |
|  |  |  |  |  |
|  |  |  |  |  |
|  |  |  |  |  |
|  |  |  |  |  |
|  |  |  |  |  |
|  |  |  |  |  |
|  | **Type of school**  1=Public (UPE/USE) day school  2=Private day school  3= Private boarding school  4= Religious day school  5= Religious boarding school  6=Other, specify_____________________________ | | **Reason for missed school days**  1=School closed  2=Child sick  3=Child needed at home (work, other)  4= Could not pay school fees or material  5=Other, specify___________________________ | |

2.3. Does the household have off-farm income (1=Yes 2=No)__________________________________________

2.4. Is the household engaged in the following pig-business related activities? (*Tick if Yes*)

| **Activity** | **Tick if yes** |
| --- | --- |
| Pig trading |  |
| Processing of pork/pork products (e.g. slaughter) |  |
| Operating a butchery |  |
| Operating a pork kiosk |  |
| Operating a pork joint |  |
| Other, specify___________________________________________ |  |

## Livestock inventory and pig herd structure flows

3.1. Indicate the type and number of livestock kept/owned currently

| **Livestock species** | **Total number owned/kept by the household** |
| --- | --- |
| Pigs |  |
| Cattle |  |
| Sheep |  |
| Goats |  |
| Poultry |  |
| Other, specify |  |

3.2. Indicate the different categories of pigs kept currently

| **Type** | **Numbers kept** | **Breed type** | **Housing** |
| --- | --- | --- | --- |
| Breeding boars |  |  |  |
| Breeding sows |  |  |  |
| Growers |  |  |  |
| Piglets |  |  |  |
| **Breed type**  1 = Local  2 = Cross  3 = Exotic | | **Housing at visit**  1= Confined  2= Tethered  3= Free range/scavenging | |

3.3. Have any pigs left your herd in the last 12 months? [ ___________ ] (1=Yes 2=No)

3.4. If yes, fill in the table below:

| **Type**  (code) | **Breed**  (code) | **How exited** (code) | **How many pigs exited?** | **In case of death or slaughter because sick** | | |
| --- | --- | --- | --- | --- | --- | --- |
|  |  |  |  | **Number of pigs that died** | **Cause of death**  (code) | **If due to disease,**  **what disease?** |
|  |  |  |  |  |  |  |
|  |  |  |  |  |  |  |
|  |  |  |  |  |  |  |
|  |  |  |  |  |  |  |
|  |  |  |  |  |  |  |
|  |  |  |  |  |  |  |
|  |  |  |  |  |  |  |
|  |  |  |  |  |  |  |
|  |  |  |  |  |  |  |
| **Type**  1 = Breeding boars  2 = Breeding sows  3 = Growers  4 = Piglets | **Breed**  1= Local  2=Cross  3= Exotic | **How exited**  1 = Sold (no specific reason)  2=Sold because sick  3=Sold because fear of pig disease  4 = Slaughter for sale  5 = Slaughter - household consumption  6=Slaughter because sick  7= Stolen  8= Death  9= Gift  10= Other, specify______________________________________________________ | | | | **In case of death, cause**  1=Disease (specify)  2=Starvation (lack of food or water)  3=Poisoned  4=Injury  5=Other, specify ____________________________ |

3.5. Has there been any inflow of pigs through purchases, births or any other form in the last 12 months? [ _________ ] (1=Yes 2=No)

3.6. If yes, please provide the details:

| **Type**  (code) | **Breed**  (code) | **Type of entry**  (code) | **No. of pigs** | **If purchased** | | | |
| --- | --- | --- | --- | --- | --- | --- | --- |
|  |  |  |  | Reason for purchase  (code) | Cost per animal (UGX) | | Purchase point  (code) |
|  |  |  |  |  |  | |  |
|  |  |  |  |  |  | |  |
|  |  |  |  |  |  | |  |
|  |  |  |  |  |  | |  |
|  |  |  |  |  |  | |  |
|  |  |  |  |  |  | |  |
|  |  |  |  |  |  | |  |
| **Type**  1 = Breeding boars  2 = Breeding sows  3 = Growers  4 = Piglets | | **Breed**  1= Local  2= Cross  3= Exotic | | **Type of entry**  1 = Bought from smallholder farm  2 = Bought from individual trader/broker  3= Bought from a large scale farm  4 = Loan from project  5 = Gift from others  6 = Birth/ born on farm  7 =Other, specify__________________________________________ | | **Reason for purchase**  1 = Replace old stock  2 = Means of saving money  3 = Prestige  4 = Expand herd  5= Other,  specify ______________ | **Purchase point**  1 = Within village  2 = Neighboring village  3 = Other, specify____________ |

3.7. When did the household start pig farming (year) [ __________________________ ]

3.8. How did you finance the different elements needed for the start-up of the pig enterprise?

| **Element** | **Amount**  (UGX) | **Source**  (Codes) |
| --- | --- | --- |
| Pigs |  |  |
| Housing |  |  |
| **Source**  1=Savings  2=Loan  3=Donation  4=Gift  5=Other, specify_______________________________________________ | | |

3.9. Have you done any expansion since the first construction? [ ________ ] (1=Yes 2=No)

3.10. Do you keep records associated with the pig enterprise? [ _________ ] (1=Yes 2=No)

3.11. If yes, what types of records do you keep?

| **Record type** | **Tick if yes** |
| --- | --- |
| Feeds |  |
| Reproduction and breeding |  |
| Animal inventory (births, deaths, sales) |  |
| Financial (income and expenditures) |  |
| Others (specify) ___________________________ |  |

## Pig related incomes

4.1. Did you sell any pigs in the last 12 months? [ _______ ] (1=Yes 2=No)

4.2. If yes, please indicate the numbers sold from each category.

| **Category** | **No. sold** | **Weight (live)** | **Weight (carcass)** | **Price/head**  (UGX) | **Sales outlet**  (code) |
| --- | --- | --- | --- | --- | --- |
| Breeding boars |  |  |  |  |  |
| Breeding sows |  |  |  |  |  |
| Growers |  |  |  |  |  |
| Piglets |  |  |  |  |  |
| **Sales outlet**  1 = Farm gate  2 = Village/local market  3 = Slaughterhouse/abattoir  4 = Butchery  5 = Others, specify_______________________________________________ | | | | | |

4.3. Did you have other incomes related to products from your own pig in the last 12 months?

[ _______ ] (1=Yes 2=No)

4.4. If yes, what was that total income in the last 12 months? (UGX) [ _________________________ ]

4.5. Do you own a breeding boar? [ __________ ] ( 1=Yes 2=No)

If no skip to 5

4.6. Do you use it/them for own or communal pig breeding? [ _______ ]

(1 = Own 2 = Village (communal) 3 = Other, specify _____________________________)

4.7. How much do you charge per service? (UGX) [ ____________________________ ]

4.8. What was your total income from the breeding boar during the last 12 months? (UGX)

[ __________________ ]

## Pig production related costs

**5.1. Breeding services**

5.1.1. Indicate the source of breeding services for the sows in the last 12 months [ _______ ]

(0= Did not do any breeding 1= Own boar 2= Other boar)

5.1.2. If you used other boars than your own, what is the cost per service? (UGX) [ ____________] if other (e.g. a piglet) specify

5.1.3. What was your total expenditure for the breeding service during the last 12 months? (UGX)

[ ________________ ]

**5.2 Animal health costs**

5.2.1 Did your pigs receive any medical treatments (deworming, antiparasitic, profylaxis, antibiotics, vaccination) in the last 12 months?

[ __________ ] (1=Yes 2=No)

5.2.2. If yes, what was your total expenditure for medical treatments during the last 12 months? (UGX) [ _______________]

5.2.3 Did you have any costs for bio security equipment (protective clothing, boots, disinfectants etc) in the last 12 months? [ __________ ] (1=Yes 2=No)

5.2.4. If yes, what was your total expenditure for bio security equipment during the last 12 months? (UGX) [ _______________]

5.2.5. Did you receive any extension services related to pigs in the last 12 months? [ ______ ]

(1=Yes 2=No)

5.2.4. If yes, what was your total expenditure for extension services related to pigs during the last 12 months (UGX)? [ _______________]

**5.3. Feeding**

5.3.1. What was your total expenditure on pig feeds during the last 12 months? (UGX) [ ___________ ]

**5.4 Labor resources**

5.4.1. Did you have any hired labour engaged in the pig enterprise during the last 12 months?

[ __________ ] (1=Yes 2=No)

5.4.2. If yes, what was your total expenditure for hired labour engaged in the pig enterprise during the last 12 months? (UGX) [ _______________]

## Household assets

6.1. Did you during the last 12 months have to sell any household assets due to losses incurred in the pig production?[ ____________ ] (1=Yes 2=No)

6.2. If yes:

| **Asset** | **Price obtained** (UGX) |
| --- | --- |
|  |  |
|  |  |
|  |  |

6.3 During the last 12 months, how many times a week did your family eat meat (on average)?_______________________

## Services

7.1. Have you needed any financial credit in the last 12 months? [ ______ ] (1=Yes 2=No)

(If No, the questionnaire is finished)

7.2. If yes, did you get the credit? [ ______ ] (1=Yes 2=No)

7.3. If no, why was credit not acquired? [ _______________ ]

(1= No collateral, 2= Credit terms unfavorable, 3=Other, specify _______________________________________)

7.4. If you needed a loan please fill in the table below (if no credit was acquired only the two first columns)

| **Reason for needing credit**  **(code)** | **Amount needed** | **Amount recieved** | **Interest rate per month** | **Use of credit** (code) |
| --- | --- | --- | --- | --- |
|  |  |  |  |  |
|  |  |  |  |  |
|  |  |  |  |  |
|  |  |  |  |  |
| **Reasons**  1=Family health problems  2=Animal health problems  3=Crop failure  4=Investments  5=Pay school fees  6=Wedding  7=Funeral  8=Other, specify_________________________________________ | | **Credit uses**  1= Feeds  2= Animal health  3= Labor  4= Capital costs  5=Others, specify________________________ | | |

**To be answered privately by the enumerator immediately following the interview**

1. In your opinion, how did you establish rapport with this respondent [____]

**1** = with ease

**2** = with some persuasion

**3** = with difficulty

**4** = it was impossible

1. Overall, how did the respondent give answers to your questions? [____]

**1** = willingly

**2** = reluctantly

**3** = with persuasion

**4** = it was hard to get answers

1. How often do you think the respondent was telling the truth? [____]

**1** = rarely

**2** = sometimes

**3** = most of the times

**4** = all the time

I certify that I have checked the questionnaire two times to be sure that all the questions have been answered, and that the answers are legible.

Signed: _______________________________________ Date ____/____/____

**Quality Assurance Aspects**

| DATE OF QUESTIONNAIRE INSPECTION BY SUPERVISOR (dd/mm/yyyy): | / / |
| --- | --- |
| Reviewing of questionnaire: | |
| **Enumerator: Enter your comments here AFTER you have administered the questionnaire** | |
| **Supervisor: Enter your comments here AFTER you have inspected the WHOLE questionnaire** | |
