# Supplementary material for: Smallholders’ perceptions on biosecurity and disease control in relation to African swine fever in an endemically infected area in Northern Uganda
Source: BMC Vet Res. 2019 Aug 5;15:279. doi: 10.1186/s12917-019-2005-7 (PMC6683333; doi:10.1186/s12917-019-2005-7)
Supplement: Supplementary file 2 — Questionnaire used in a study conducted with smallholder pig-farmers in northern Uganda 2014–2015. Second interview. (DOCX 30 kb) [file 12917_2019_2005_MOESM2_ESM.docx]

| **1. Questionnaire ID** |
| --- |

| ___________ |
| --- |

| **2. Date of Survey** |
| --- |

| ❑ | 2014-09-17 |
| --- | --- |
| ❑ | 2014-09-18 |
| ❑ | 2014-09-19 |
| ❑ | 2014-09-20 |
| ❑ | 2014-09-21 |
| ❑ | 2014-09-22 |
| ❑ | 2014-09-23 |
| ❑ | 2014-09-24 |
| ❑ | 2014-09-25 |
| ❑ | 2014-09-26 |
| ❑ | 2014-09-27 |
| ❑ | 2014-09-28 |
| ❑ | 2014-09-29 |
| ❑ | 2014-09-30 |
| ❑ | 2014-10-01 |
| ❑ | 2014-10-02 |
| ❑ | 2014-10-03 |
| ❑ | 2014-10-04 |
| ❑ | 2014-10-05 |
| ❑ | 2014-10-06 |
| ❑ | 2014-10-07 |
| ❑ | 2014-10-08 |
| ❑ | 2014-10-09 |

| **3. You participated in a previous part of this project by answering many questions about you and your pigs. According to you, how many months has passed since we here last time?** |
| --- |

| ❑ | 1 |
| --- | --- |
| ❑ | 2 |
| ❑ | 3 |
| ❑ | 4 |
| ❑ | 5 |
| ❑ | 6 |
| ❑ | 7 |
| ❑ | 8 |
| ❑ | 9 |
| ❑ | 10 |
| ❑ | 11 |
| ❑ | 12 |

| **4. Enumerator** |
| --- |

| ❑ | Alike Solomon |
| --- | --- |
| ❑ | Bruce Nokorach |
| ❑ | Peter Ogweng |

| **5. Time interview started** |
| --- |

| ______________________________ |
| --- |

| **6. Time intreview ended** |
| --- |

| ______________________________ |
| --- |

| **7. Name of the head of the household** |
| --- |

| ______________________________ |
| --- |

| **8. Respondents name** |
| --- |

| ______________________________ |
| --- |

| **9. Respondents telephone number** |
| --- |

| ______________________________ |
| --- |

| **10. Gender of respondent** |
| --- |

| ❑ | Male |
| --- | --- |
| ❑ | Female |

| **11. Marital status of household head** |
| --- |

| ❑ | Married |
| --- | --- |
| ❑ | Widow/widower |
| ❑ | Single parent |
| ❑ | Other (specify) |

| If other, specify: |
| --- |
|  |
| ______________________________ |

| **12. Subcounty** |
| --- |

| ❑ | Awach |
| --- | --- |
| ❑ | Bardege |
| ❑ | Bobi |
| ❑ | Bungatira |
| ❑ | Koro |
| ❑ | Lakwana |
| ❑ | Lalogi |
| ❑ | Odek |
| ❑ | Ongako |
| ❑ | Paicho |
| ❑ | Palaro |
| ❑ | Patiko |
| ❑ | Unyama |

| **13. Parish** |
| --- |

| ❑ | Acoyo |
| --- | --- |
| ❑ | Abwoch |
| ❑ | Agonga |
| ❑ | Alokolum |
| ❑ | Angaya |
| ❑ | Atiabar |
| ❑ | Bardege |
| ❑ | Binya |
| ❑ | Forgod |
| ❑ | Gem |
| ❑ | Gweng Diya |
| ❑ | Ibakara |
| ❑ | Idobo |
| ❑ | Kal |
| ❑ | Kal-ali |
| ❑ | Kalumu |
| ❑ | Kanyagoga |
| ❑ | Kasubi |
| ❑ | Labworomor |
| ❑ | Laliya |
| ❑ | Lamola |
| ❑ | Lapinat west |
| ❑ | Laroo |
| ❑ | Lujorogole |
| ❑ | Lukwir |
| ❑ | Mede |
| ❑ | Otino |
| ❑ | Pabwo |
| ❑ | Paduny |
| ❑ | Paidwe |
| ❑ | Pakwelo |
| ❑ | Palenga |
| ❑ | Parwech |
| ❑ | Patuda |
| ❑ | Pawel |
| ❑ | Pugwinyi |
| ❑ | Pukony |
| ❑ | Te-got |

| **14. Village** |
| --- |

| ______________________________ |
| --- |

| **15. GPS coordinates Latitdues N/S** |
| --- |

| ___________ |
| --- |

| **16. GPS coordinates Longitudes E/W** |
| --- |

| ___________ |
| --- |

| **17. Household details: Did anyone leave or enter the household since last visist?** |
| --- |

| Compare with list from last visit |
| --- |

| ❑ | Yes |
| --- | --- |
| ❑ | No |

| Specify if entry or exit, if entry provide details in question below, if exit specify whom. Compare to list from last visit. |
| --- |
|  |
|  |
|  |

| **18. Household details** |
| --- |

| Gender: 1=Male, 2=Female  Relationship to household head:  1 = Head, 2 = Spouse , 3 = Child, 4 = Sibling, 5 = Parent,  6 = Grandchild, 7 = Other relative, 8 = Non-relative (including employees who live in house), 9 = Other (specify in comments)  Highest education level:  0 = No formal education, 1 = Nursery, 2 = Pre-school age,  3 = Primary education (P1-P4),  4 = Primary education (P5-P7), 5 = Secondary school (S1-S2),  6 = Secondary school (S3-S4),  7 = High school (S5-S6), 8 = Vocational training (specify no of years in comments), 9 = Tertiary training (specify no of years in comments), 10 = University degree (undergraduate)  11 = University degree (postgraduate), 12=Adult literacy, 13=Other (specify in comments)  Primary source of income:  0 = None, 1 = Crop farming, 2 = Pig keeping (incl. sales) , 3 = Cattle keeping, 4 = Poultry/keeping (inc. sales), 5 = Salaried employment, 6 = Self-employed-off farm, 7 = Casual laborer, 8 = Boda boda,  9 = Student/pupil, 10 = Charcoal burning, 11 = Pre-school age,  12 = Other (specify in comments)   \|  \| **Members of household**  [*FIRST NAMES]* \| **Year of birth** \| **Gender** \| **Relationship to household head** \| **Highest education level attained** \| **Primary source of income** \| \| --- \| --- \| --- \| --- \| --- \| --- \| --- \| \| 1 \|  \|  \|  \|  \|  \|  \| \| 2 \|  \|  \|  \|  \|  \|  \| \| 3 \|  \|  \|  \|  \|  \|  \| \| 4 \|  \|  \|  \|  \|  \|  \| |
| --- | --- | --- | --- | --- | --- | --- | --- | --- | --- | --- | --- | --- | --- | --- | --- | --- | --- | --- | --- | --- | --- | --- | --- | --- | --- | --- | --- | --- | --- | --- | --- | --- | --- | --- | --- |
| **Comments** |

| **20. Chidren of school age:** |
| --- |

| Type of school:  1=Public (UPE/USE) day school, 2=Private day school, 3= Private boarding school, 4= Religious day school,  5= Religious boarding school, 6=Other (specify in comments)  Reason for missed school days:  1=School closed, 2=Child sick, 3=Child needed at home (work, other), 4= Could not pay school fees or material, 5=Other (specify in comments) |
| --- |

|  | **Name** | **Type of school** | **Cost per term** | **Number of missed schooldays during last term** | **Reason for**  **missed**  **schooldays** |
| --- | --- | --- | --- | --- | --- |
| 1 |  |  |  |  |  |
| 2 |  |  |  |  |  |
| 3 |  |  |  |  |  |
| 4 |  |  |  |  |  |
| 5 |  |  |  |  |  |
| 6 |  |  |  |  |  |
| 7 |  |  |  |  |  |
| 8 |  |  |  |  |  |
| 9 |  |  |  |  |  |
| 10 |  |  |  |  |  |
| 11 |  |  |  |  |  |
| 12 |  |  |  |  |  |
| 13 |  |  |  |  |  |
| 14 |  |  |  |  |  |
| 15 |  |  |  |  |  |

|  |  |  |  |  |  | |
| --- | --- | --- | --- | --- | --- | --- |
|  |  |  |  |  |  | |
| **Comments** | | | | | |  |
| **22. Does the household have off-farm income?** | | | | | |  |

| ❑ | Yes |
| --- | --- |
| ❑ | No |

| **23. Is the household engaged in the following pig related activities** |
| --- |

| ❑ | Pig trading |
| --- | --- |
| ❑ | Processing of pork/pork products (e.g. slaughter) |
| ❑ | Operating a butchery |
| ❑ | Operating a pork kiosk |
| ❑ | Operating a pork joint |
| ❑ | Other |

| If other, specify |
| --- |
|  |
| ______________________________ |

| **24. Indicate the type and number of livestock kept/owned currently** |
| --- |

| Pigs | _________________________ |
| --- | --- |
| Cattle | _________________________ |
| Sheep | _________________________ |
| Goats | _________________________ |
| Poultry | _________________________ |
| Other | _________________________ |

| **25. Indicate the different categories of pigs kept currently:** |
| --- |

| Breed type: 1=Local, 2=Cross, 3=Exotic  Housing: 1=Confined, 2=Tethered, 3=Free range |
| --- |

|  | Numbers kept | Breed type | Housing |
| --- | --- | --- | --- |
| Breeding boars | ___________ | ___________ | ___________ |
| Breeding sows | ___________ | ___________ | ___________ |
| Growers | ___________ | ___________ | ___________ |
| Piglets | ___________ | ___________ | ___________ |

| **26. Have any pigs left your herd since the last visit?** |
| --- |

| ❑ | Yes |
| --- | --- |
| ❑ | No |

| **27. Pig exits** |
| --- |

| Breed: 1=Local, 2=Cross, 3=Exotic  How exited: 1=Sold, 2=Sold because sick, 3=Sold because fear of pig disease, 4=Slaughter for sale,  5= Slaughter for household consumption, 6=Slaughter because sick, 7=Stolen, 8=Death, 9=Gift, 10=Other (specify in comments)  In case of death, cause: 1=Disease, 2=Starvation, 3=Poisoned, 4=Injury, 5=Other (specify in comments) |
| --- |

|  | **Breed** | **How exited** | **How many pigs exited** | **How many pigs died** | **In case of death; cause** | **If disease; which** |
| --- | --- | --- | --- | --- | --- | --- |
| Breeding boars |  |  |  |  |  |  |
| Breeding boars |  |  |  |  |  |  |
| Breeding boars |  |  |  |  |  |  |
| Breeding sows |  |  |  |  |  |  |
| Breeding sows |  |  |  |  |  |  |
| Breeding sows |  |  |  |  |  |  |
| Growers |  |  |  |  |  |  |
| Growers |  |  |  |  |  |  |
| Growers |  |  |  |  |  |  |
| Piglets |  |  |  |  |  |  |
| Piglets |  |  |  |  |  |  |
| Piglets |  |  |  |  |  |  |

| 28. Comments |
| --- |

|  |
| --- |
|  |
|  |
|  |

| **29. Has there been any inflow of pigs through purchases, births or any other form since the last visit?** |
| --- |

| ❑ | Yes |
| --- | --- |
| ❑ | No |

| **30. Pig entries** |
| --- |

| Breed: 1=Local, 2=Cross, 3=Exotic  Type of entry: 1=Bought from smallholder farm, 2=Bought from individual trader/broker, 3=Bought from a large scale farm, 4=Loan from project, 5=Gift, 6=Birth/born on farm, 7=Other (specify in comments)  Reason for purchase: 1=Replace old stock, 2=Saving money, 3=Prestige, 4=Expand herd, 5=Other (specify in commetns)  Purchase point: 1=Within village, 2=Neighbouring village, 3=Other (specify in comments) |
| --- |

| \|  \| **Breed** \| **Type of entry** \| **How many pigs** \| **Reason for purchase** \| **Cost per animal** \| **Purchase point** \| \| --- \| --- \| --- \| --- \| --- \| --- \| --- \| \| Breeding boars \|  \|  \|  \|  \|  \|  \| \| Breeding boars \|  \|  \|  \|  \|  \|  \| \| Breeding boars \|  \|  \|  \|  \|  \|  \| \| Breeding sows \|  \|  \|  \|  \|  \|  \| \| Breeding sows \|  \|  \|  \|  \|  \|  \| \| Breeding sows \|  \|  \|  \|  \|  \|  \| \| Growers \|  \|  \|  \|  \|  \|  \| \| Growers \|  \|  \|  \|  \|  \|  \| \| Growers \|  \|  \|  \|  \|  \|  \| \| Piglets \|  \|  \|  \|  \|  \|  \| \| Piglets \|  \|  \|  \|  \|  \|  \| \| Piglets \|  \|  \|  \|  \|  \|  \|   **31. Comments** |
| --- | --- | --- | --- | --- | --- | --- | --- | --- | --- | --- | --- | --- | --- | --- | --- | --- | --- | --- | --- | --- | --- | --- | --- | --- | --- | --- | --- | --- | --- | --- | --- | --- | --- | --- | --- | --- | --- | --- | --- | --- | --- | --- | --- | --- | --- | --- | --- | --- | --- | --- | --- | --- | --- | --- | --- | --- | --- | --- | --- | --- | --- | --- | --- | --- | --- | --- | --- | --- | --- | --- | --- | --- | --- | --- | --- | --- | --- | --- | --- | --- | --- | --- | --- | --- | --- | --- | --- | --- | --- | --- | --- |

| ______________________________ |
| --- |
| ______________________________ |
| ______________________________ |
| ______________________________ |

| **32. Have you done any expansion in the pig enterprise since last visit?** |
| --- |

| ❑ | Yes |
| --- | --- |
| ❑ | No |

| **33. If yes, specify how:** |
| --- |

| ______________________________ |
| --- |
| ______________________________ |

| **34. Do you keep records associated with the pig enterprise?** |
| --- |

| ❑ | Yes |
| --- | --- |
| ❑ | No |

| **35. What types of records?** |
| --- |

| ❑ | Feeds |
| --- | --- |
| ❑ | Reproduction and breeding |
| ❑ | Animal inventory (births, deaths, sales) |
| ❑ | Financial (income and expenditure) |
| ❑ | Other |

| If other, specify |
| --- |
|  |
| ______________________________ |

| **36. Did you sell any pigs since the last visit?** |
| --- |

| ❑ | Yes |
| --- | --- |
| ❑ | No |

| **37. Indicate the numbers sold from each pig category:** |
| --- |

| **Sales outlet: 1=Farm gate, 2=Village/local market. 3=Slaughterhouse/abbatoir, 4=Butchery, 5=Other (specify in coments)** |
| --- |

| \|  \| **How many sold** \| **Weight (live)** \| **Weight (carcass)** \| **Price/head (UGX)** \| **Sales outlet** \| \| --- \| --- \| --- \| --- \| --- \| --- \| \| Breeding boars \|  \|  \|  \|  \|  \| \| Breeding sows \|  \|  \|  \|  \|  \| \| Growers \|  \|  \|  \|  \|  \| \| Piglets \|  \|  \|  \|  \|  \|   **38. Comments** |
| --- | --- | --- | --- | --- | --- | --- | --- | --- | --- | --- | --- | --- | --- | --- | --- | --- | --- | --- | --- | --- | --- | --- | --- | --- | --- | --- | --- | --- | --- | --- |

| ______________________________ |
| --- |
| ______________________________ |
| ______________________________ |
| ______________________________ |

| **39. Did you have any other income related to products from your own pigs since the last visit?** |
| --- |

| ❑ | Yes |
| --- | --- |
| ❑ | No |

| **40. If yes, what was the total income since the last visit?** |
| --- |

| ______________________________ |
| --- |

| **41. Do you own a breeding boar?**  **(If no skip to Q 44)** |
| --- |

| ❑ | Yes |
| --- | --- |
| ❑ | No |

| **42. Do you use it/them for own or communal breeding?** |
| --- |

| ❑ | Own |
| --- | --- |
| ❑ | Village/communal |
| ❑ | Other |

| If other, specify: |
| --- |
|  |
| ______________________________ |

| **43. How much do you charge per service (UGX)?** |
| --- |

| _________________________________________________________________ |
| --- |

| **44. What was your total income from the breeding boar since the last visit?** |
| --- |

| ______________________________ |
| --- |

| **45. Indicate the source of breeding for the sows since the last visit** |
| --- |

| ❑ | Didnt do any breeding |
| --- | --- |
| ❑ | Own boar |
| ❑ | Other boar |

| If other, specify: |
| --- |
|  |
| ______________________________ |

| **46. What is the cost per service (UGX or other)?** |
| --- |

| ______________________________ |
| --- |

| **47. What was your total expenditure for the breeding service since the last visit?** |
| --- |

| ______________________________ |
| --- |

| **48. Did you have any hired labour engaged in the pig enterprise since the last visit?** |
| --- |

| ❑ | Yes |
| --- | --- |
| ❑ | No |

| **49. If yes, what was your total expenditure for hired labour engaged in the pig enterprise since the last visit (UGX)?** |
| --- |

| ______________________________ |
| --- |

| **50. Did your pigs recieve any medical treatments (deworming, antiparasitic, profylaxis, antibiotics, vaccination) since the last visit?** |
| --- |

| ❑ | Yes |
| --- | --- |
| ❑ | No |

| **51. If yes, what treatment(s)?** |
| --- |

| ______________________________ |
| --- |
| ______________________________ |

| **52. What was your total expenditure for medical treatments since the last visit (UGX )?** |
| --- |

| ______________________________ |
| --- |

| **53. Did you have any expenditure for biosecurity eqipment (protective clothing, boots, disinfectants etc) since the last visit?** |
| --- |

| ❑ | Yes |
| --- | --- |
| ❑ | No |

| **54. If yes, what sort fo equipment did you buy?** |
| --- |

| ______________________________ |
| --- |
| ______________________________ |

| **55. What was your total expenditure for bio security eqipment since the last visit (UGX )?** |
| --- |

| ______________________________ |
| --- |

| **56. Did you receive any extension service related to pigs since the last visit?** |
| --- |

| ❑ | Yes |
| --- | --- |
| ❑ | No |

| **57. What was your total expenditure for extension service related to pigs since the last visit (UGX)?** |
| --- |

| ______________________________ |
| --- |

| **58. What was your total expenditure on pig feeds since the last visit (UGX)?** |
| --- |

| ______________________________ |
| --- |

| **59. Since the last visit, did you have to sell any household assets due to losses incured in the pig production?** |
| --- |

| ❑ | Yes |
| --- | --- |
| ❑ | No |

| **60. If yes, Indicate what asset and the price obtained** |
| --- |

|  | Type of asset | Price obtained |
| --- | --- | --- |
| Asset | ___________ | ___________ |
| Asset | ___________ | ___________ |
| Asset | ___________ | ___________ |

| **61. Since the last visit, how many times a week did your family eat meat (on average)?** |
| --- |

| ______________________________ |
| --- |

| **62. Have you needed any financial credit since the last visit)?** |
| --- |

| ❑ | Yes |
| --- | --- |
| ❑ | No |

| **63. If yes, did you get the credit?** |
| --- |

| ❑ | Yes |
| --- | --- |
| ❑ | No |

| **64. If no, why was credit not acquired?** |
| --- |

| ❑ | No collateral |
| --- | --- |
| ❑ | Credit terms unfavourable |
| ❑ | Other |

| If other, specify: |
| --- |
|  |
| ______________________________ |

| **65. Amonut needed, recieved, interest rate and use of credit** |
| --- |

| Reasons: 1=Family health problems, 2=Animal health problems, 3=Crop failure, 4=Investments, 5=Pay school fees, 6=Wedding, 7=Funeral, 8= Other, spcify in comments  Use of credit: 1=Feeds, 2=Animal health, 3=Labour, 4=Capital costs, 5=Other (specify in comments) |
| --- |

| \|  \| **Reason for needing credit** \| **Amount needed** \| **Amount received** \| **Interest rate** \| **Use of credit** \| \| --- \| --- \| --- \| --- \| --- \| --- \| \| Credit 1 \|  \|  \|  \|  \|  \| \| Credit 2 \|  \|  \|  \|  \|  \| \| Credit 3 \|  \|  \|  \|  \|  \|   **66. Comments** |
| --- | --- | --- | --- | --- | --- | --- | --- | --- | --- | --- | --- | --- | --- | --- | --- | --- | --- | --- | --- | --- | --- | --- | --- | --- |

| ______________________________ |
| --- |
| ______________________________ |

| **67. Since the last visit;** **67. Since the last visit;**     \|  \| No, none \| Most not \| Some yes, some not \| Yes, most \| Yes, all \| \| --- \| --- \| --- \| --- \| --- \| --- \| \| Have the family been able to pay all needed school fees? \|  \|  \|  \|  \|  \| \| Have the family been able to meet all medical expenses that has come up \|  \|  \|  \|  \|  \| \| Have there been any family gatherings (weddings, funeral, baptisms) etc that had to be changed or postponed due to lack of money? \|  \|  \|  \|  \|  \| |
| --- | --- | --- | --- | --- | --- | --- | --- | --- | --- | --- | --- | --- | --- | --- | --- | --- | --- | --- | --- | --- | --- | --- | --- | --- |

|  |  |  |  |  |  |  |
| --- | --- | --- | --- | --- | --- | --- |
|  |  |  |  |  |  |  |
|  |  |  |  |  |  |  |
|  |  |  |  |  |  |  |

| **Comments** |
| --- |
|  |
| **______________________________** |
| **______________________________** |

| **68. Since the last visit;**   \|  \| No, never \| Most of the times not \| Sometimes yes, sometimes not \| Yes, most of the time \| Yes, always \| \| --- \| --- \| --- \| --- \| --- \| --- \| \| I feel more optimistic about the pig enterprise \|  \|  \|  \|  \|  \| \| There has been an increase in disputes, disagreements or jealousy among my neighbours \|  \|  \|  \|  \|  \| \| I have lost confidence in pig production \|  \|  \|  \|  \|  \| \| I am no longer participating in the social networks like I used to do \|  \|  \|  \|  \|  \|   **Comments**   \| **______________________________** \| \| --- \| \| **______________________________** \|  \| **69. How do you agree with the following statements;**   \|  \| Strongly disagree \| Disagree \| Neither agree nor disagree \| Agree \| Strongly agree \| \| --- \| --- \| --- \| --- \| --- \| --- \| \| I think it is possible to protect my pigs from getting ASF by improving farm bio security \|  \|  \|  \|  \|  \| \| Eating pork from pigs that have died from ASF is safe for human health \|  \|  \|  \|  \|  \| \| If I would get a fair price I would be willing to sell all my heatlhy pigs when an ASF-outbreak were present in the area \|  \|  \|  \|  \|  \| \| I would like to invest in farm bio security if I recieved advice on what to do \|  \|  \|  \|  \|  \| \| I would be happy to buy pork products from a slaughterhouse that recieve pigs that have been in contact with pigs dying from ASF \|  \|  \|  \|  \|  \| \| It is safe to give pigs water that has been used to clean knifes and pangas used for slasughtering and butchering as drinking water \|  \|  \|  \|  \|  \| \| Buying live pigs is a risk behaviour for contracting ASF \|  \|  \|  \|  \|  \| \| I dont want to eat or buy pork from pigs that have died from ASF \|  \|  \|  \|  \|  \| \| I can not afford to invest in my pig farming \|  \|  \|  \|  \|  \| \| ASF can not be prevented \|  \|  \|  \|  \|  \| \| I can choose where/to whom I sell my pigs \|  \|  \|  \|  \|  \| \| Frequent sellling and buying of pigs is neccessary for succesfull pig farming \|  \|  \|  \|  \|  \| \| Improved farm bio security improves pig health and pig growth \|  \|  \|  \|  \|  \| \| I could adopt my pig farming in order to have pigs ready for sale at specific times of the year \|  \|  \|  \|  \|  \| \| Cooking kills the ASF-virus \|  \|  \|  \|  \|  \| \| It is possible for me to tell visitors such as veterinarians, middle men and extension workers not to enter in the pig house with their own boots \|  \|  \|  \|  \|  \| \| If pork prices are lower in the neighbouring village due to them having an outbreak of ASF I will buy my pork there \|  \|  \|  \|  \|  \|  \| **Comments** \| \| --- \| \|  \| \| ______________________________ \| \| ______________________________ \|  \| **70. Comments** \| \| --- \|  \| ______________________________ \| \| --- \| \| ______________________________ \| \| ______________________________ \| \| ______________________________ \| \| \| --- \| --- \| --- \| --- \| --- \| --- \| --- \| --- \| --- \| --- \| --- \| --- \| --- \| --- \| --- \| --- \| --- \| --- \| --- \| --- \| --- \| --- \| --- \| --- \| --- \| --- \| --- \| --- \| --- \| --- \| --- \| --- \| --- \| --- \| --- \| --- \| --- \| --- \| --- \| --- \| --- \| --- \| --- \| --- \| --- \| --- \| --- \| --- \| --- \| --- \| --- \| --- \| --- \| --- \| --- \| --- \| --- \| --- \| --- \| --- \| --- \| --- \| --- \| --- \| --- \| --- \| --- \| --- \| --- \| --- \| --- \| --- \| --- \| --- \| --- \| --- \| --- \| --- \| --- \| --- \| --- \| --- \| --- \| --- \| --- \| --- \| --- \| --- \| --- \| --- \| --- \| --- \| --- \| --- \| --- \| --- \| --- \| --- \| --- \| --- \| --- \| --- \| --- \| --- \| --- \| --- \| --- \| --- \| --- \| --- \| --- \| --- \| --- \| --- \| --- \| --- \| --- \| --- \| |
| --- | --- | --- | --- | --- | --- | --- | --- | --- | --- | --- | --- | --- | --- | --- | --- | --- | --- | --- | --- | --- | --- | --- | --- | --- | --- | --- | --- | --- | --- | --- | --- | --- | --- | --- | --- | --- | --- | --- | --- | --- | --- | --- | --- | --- | --- | --- | --- | --- | --- | --- | --- | --- | --- | --- | --- | --- | --- | --- | --- | --- | --- | --- | --- | --- | --- | --- | --- | --- | --- | --- | --- | --- | --- | --- | --- | --- | --- | --- | --- | --- | --- | --- | --- | --- | --- | --- | --- | --- | --- | --- | --- | --- | --- | --- | --- | --- | --- | --- | --- | --- | --- | --- | --- | --- | --- | --- | --- | --- | --- | --- | --- | --- | --- | --- | --- | --- | --- | --- | --- | --- | --- | --- | --- | --- | --- | --- | --- | --- | --- | --- | --- | --- | --- | --- | --- | --- | --- | --- | --- | --- | --- | --- | --- | --- | --- | --- | --- | --- | --- | --- |
