# Supplementary material for: Smallholders’ perceptions on biosecurity and disease control in relation to African swine fever in an endemically infected area in Northern Uganda
Source: BMC Vet Res. 2019 Aug 5;15:279. doi: 10.1186/s12917-019-2005-7 (PMC6683333; doi:10.1186/s12917-019-2005-7)
Supplement: Supplementary file 3 — Questionnaire used in a study conducted with smallholder pig-farmers in northern Uganda 2014–2015. Third interview. (DOC 250 kb) [file 12917_2019_2005_MOESM3_ESM.doc]

| **1. Questionnaire ID**________________________________ |
| --- |

| **2. Date of Survey** |
| --- |

| ❑ | 2015-02-18 |
| --- | --- |
| ❑ | 2015-02-19 |
| ❑ | 2015-02-20 |
| ❑ | 2015-02-21 |
| ❑ | 2015-02-24 |
| ❑ | 2015-02-25 |
| ❑ | 2015-02-26 |
| ❑ | 2015-02-27 |
| ❑ | 2015-02-28 |
| ❑ | 2015-03-01 |
| ❑ | 2015-03-02 |
| ❑ | 2015-03-03 |
| ❑ | 2015-03-04 |
| ❑ | 2015-03-05 |
| ❑ | 2015-03-06 |
| ❑ | 2015-03-07 |

| **3. You have participated in two previous part of this project by answering many questions about you and your pigs. According to you, how many months has passed since we here last time?** |
| --- |

| ❑ | 1 |
| --- | --- |
| ❑ | 2 |
| ❑ | 3 |
| ❑ | 4 |
| ❑ | 5 |
| ❑ | 6 |
| ❑ | 7 |
| ❑ | 8 |
| ❑ | 9 |
| ❑ | 10 |
| ❑ | 11 |
| ❑ | 12 |

| **4. Enumerator** |
| --- |

| ❑ | Alike Solomon |
| --- | --- |
| ❑ | Bruce Nokorach |

| **5. Time interview started**___________________ |
| --- |

| **6. Time interview ended**____________________ |
| --- |

| **7. Name of the head of the household** |
| --- |

| ___________________________________________________ |
| --- |

| **8. Respondents name** |
| --- |

| ______________________________ |
| --- |

| **9. Respondents telephone number** |
| --- |

| ______________________________ |
| --- |

| **10. Gender of respondent** |
| --- |

| ❑ | Male |
| --- | --- |
| ❑ | Female |

| **11. Marital status of household head** |
| --- |

| ❑ | Married |
| --- | --- |
| ❑ | Widow/widower |
| ❑ | Single parent |
| ❑ | Other (specify) |

| If other, specify:_________________________________________ |
| --- |
|  |
| **12. Subcounty** |

| ❑ | Awach |
| --- | --- |
| ❑ | Bardege |
| ❑ | Bobi |
| ❑ | Bungatira |
| ❑ | Koro |
| ❑ | Lakwana |
| ❑ | Lalogi |
| ❑ | Odek |
| ❑ | Ongako |
| ❑ | Paicho |
| ❑ | Palaro |
| ❑ | Patiko |
| ❑ | Unyama |

| **13. Parish** |
| --- |

| ❑ | Acoyo |
| --- | --- |
| ❑ | Abwoch |
| ❑ | Agonga |
| ❑ | Alokolum |
| ❑ | Angaya |
| ❑ | Atiabar |
| ❑ | Bardege |
| ❑ | Binya |
| ❑ | Forgod |
| ❑ | Gem |
| ❑ | Gweng Diya |
| ❑ | Ibakara |
| ❑ | Idobo |
| ❑ | Kal |
| ❑ | Kal-ali |
| ❑ | Kalumu |
| ❑ | Kanyagoga |
| ❑ | Kasubi |
| ❑ | Labworomor |
| ❑ | Laliya |
| ❑ | Lamola |
| ❑ | Lapinat west |
| ❑ | Laroo |
| ❑ | Lujorogole |
| ❑ | Lukwir |
| ❑ | Mede |
| ❑ | Otino |
| ❑ | Pabwo |
| ❑ | Paduny |
| ❑ | Paidwe |
| ❑ | Pakwelo |
| ❑ | Palenga |
| ❑ | Parwech |
| ❑ | Patuda |
| ❑ | Pawel |
| ❑ | Pugwinyi |
| ❑ | Pukony |
| ❑ | Te-got |

| **14. Village**__________________________________________ |
| --- |

| **15. GPS coordinates Latitdues N/S**_______________________ |
| --- |

| **16. GPS coordinates Longitudes E/W**_____________________ |
| --- |

| **17. Household details: Did anyone leave or enter the household since last visit?** |
| --- |

| Compare with list from last visit |
| --- |

| ❑ | Yes |
| --- | --- |
| ❑ | No |

| Specify if entry or exit, if entry provide details in question below, if exit specify whom:_________________________________________ |
| --- |
|  |
| **18. Household details** |

| Gender: 1=Male, 2=Female  Relationship to household head:  1 = Head, 2 = Spouse , 3 = Child, 4 = Sibling, 5 = Parent,  6 = Grandchild, 7 = Other relative, 8 = Non-relative (including employees who live in house), 9 = Other (specify in comments)  Highest education level:  0 = No formal education, 1 = Nursery, 2 = Pre-school age, 3 = Primary education (P1-P4),  4 = Primary education (P5-P7), 5 = Secondary school (S1-S2), 6 = Secondary school (S3-S4),  7 = High school (S5-S6), 8 = Vocational training (specify no of years in comments),  9 = Tertiary training (specify no of years in comments), 10 = University degree (undergraduate)  11 = University degree (postgraduate), 12=Adult literacy, 13=Other (specify in comments)  Primary source of income:  0 = None, 1 = Crop farming, 2 = Pig keeping (incl. sales),  3 = Cattle keeping,  4 = Poultry/keeping (inc. sales), 5 = Salaried employment, 6 = Self-employed-off farm, 7 = Casual laborer, 8 = Boda boda, 9 = Student/pupil, 10 = Charcoal burning, 11 = Pre-school age, 12 = Other (specify in comments) | | | | | | |  |
| --- | --- | --- | --- | --- | --- | --- | --- |
|  |  | **Year of birth** | **Gender** | **Relationship to household head** | **Highest education level attained** | **Primary source of income** | |
| 1 |  |  |  |  |  |  | |
| 2 |  |  |  |  |  |  | |
| 3 |  |  |  |  |  |  | |
| 4 |  |  |  |  |  |  | |

| **19. Comments** |
| --- |

|  |
| --- |
|  |
| **20. Total number of household members:**____________ |
|  |

| **21. Chidren of school age:** |
| --- |

| Type of school:  1=Public (UPE/USE) day school, 2=Private day school, 3= Private boarding school, 4= Religious day school, 5= Religious boarding school, 6=Other (specify in comments)  Reason for missed school days:  1=School closed, 2=Child sick, 3=Child needed at home (work, other), 4= Could not pay school fees or material, 5=Other (specify in comments) |
| --- |

|  | **Name** | **Type of school** | **Cost per term** | **Number of missed schooldays during last term** | **Reason for**  **missed**  **schooldays** |
| --- | --- | --- | --- | --- | --- |
| 1 |  |  |  |  |  |
| 2 |  |  |  |  |  |
| 3 |  |  |  |  |  |
| 4 |  |  |  |  |  |
| 5 |  |  |  |  |  |
| 6 |  |  |  |  |  |
| 7 |  |  |  |  |  |
| 8 |  |  |  |  |  |
| 9 |  |  |  |  |  |
| 10 |  |  |  |  |  |
| 11 |  |  |  |  |  |
| 12 |  |  |  |  |  |
| 13 |  |  |  |  |  |
| 14 |  |  |  |  |  |
| 15 |  |  |  |  |  |

| **22. Comments** |
| --- |

| **23. Does the household have off-farm income?** |
| --- |

| ❑ | Yes |
| --- | --- |
| ❑ | No |

| **24. Is the household engaged in the following pig related activities** |
| --- |

| ❑ | Pig trading |
| --- | --- |
| ❑ | Processing of pork/pork products (e.g. slaughter) |
| ❑ | Operating a butchery |
| ❑ | Operating a pork kiosk |
| ❑ | Operating a pork joint |
| ❑ | Other |

| If other, specify:___________________________________________ |
| --- |
|  |
| **25. Indicate the type and number of livestock kept/owned currently** |

| Pigs | _________________________ |
| --- | --- |
| Cattle | _________________________ |
| Sheep | _________________________ |
| Goats | _________________________ |
| Poultry | _________________________ |
| Other | _________________________ |

| **26. Indicate the different categories of pigs kept currently:** |
| --- |

| Breed type: 1=Local, 2=Cross, 3=Exotic  Housing: 1=Confined, 2=Tethered, 3=Free range |
| --- |

|  | Numbers kept | Breed type | Housing |
| --- | --- | --- | --- |
| Breeding boars | ___________ | ___________ | ___________ |
| Breeding sows | ___________ | ___________ | ___________ |
| Growers | ___________ | ___________ | ___________ |
| Piglets | ___________ | ___________ | ___________ |

| **27. Have any pigs left your herd since the last visit?** |
| --- |

| ❑ | Yes |
| --- | --- |
| ❑ | No |

| **28. Pig exits** |
| --- |
| Breed: 1=Local, 2=Cross, 3=Exotic  How exited: 1=Sold, 2=Sold because sick, 3=Sold because fear of pig disease, 4=Slaughter for sale, 5= Slaughter for household consumption, 6=Slaughter because sick, 7=Stolen, 8=Death, 9=Gift, 10=Other (specify in comments)  In case of death, cause: 1=Disease, 2=Starvation, 3=Poisoned, 4=Injury, 5=Other (specify in comments) |

|  | **Breed** | **How exited** | **How many pigs exited** | **How many pigs died** | **In case of death; cause** | **If disease; which** |
| --- | --- | --- | --- | --- | --- | --- |
| Breeding boars |  |  |  |  |  |  |
| Breeding boars |  |  |  |  |  |  |
| Breeding boars |  |  |  |  |  |  |
| Breeding sows |  |  |  |  |  |  |
| Breeding sows |  |  |  |  |  |  |
| Breeding sows |  |  |  |  |  |  |
| Growers |  |  |  |  |  |  |
| Growers |  |  |  |  |  |  |
| Growers |  |  |  |  |  |  |
| Piglets |  |  |  |  |  |  |
| Piglets |  |  |  |  |  |  |
| Piglets |  |  |  |  |  |  |

| **29. Comments** |
| --- |

| **30. Has there been any inflow of pigs through purchases, births or any other form since the last visit?** |
| --- |

| ❑ | Yes |
| --- | --- |
| ❑ | No |

| **31. Pig entries** |
| --- |
| Breed: 1=Local, 2=Cross, 3=Exotic  Type of entry: 1=Bought from smallholder farm, 2=Bought from individual trader/broker, 3=Bought from a large scale farm, 4=Loan from project, 5=Gift, 6=Birth/born on farm, 7=Other (specify in comments)  Reason for purchase: 1=Replace old stock, 2=Saving money, 3=Prestige, 4=Expand herd, 5=Other (specify in commetns)  Purchase point: 1=Within village, 2=Neighbouring village, 3=Other (specify in comments) |

|  | **Breed** | **Type of entry** | **How many pigs** | **Reason for purchase** | **Cost per animal** | **Purchase point** |
| --- | --- | --- | --- | --- | --- | --- |
| Breeding boars |  |  |  |  |  |  |
| Breeding boars |  |  |  |  |  |  |
| Breeding boars |  |  |  |  |  |  |
| Breeding sows |  |  |  |  |  |  |
| Breeding sows |  |  |  |  |  |  |
| Breeding sows |  |  |  |  |  |  |
| Growers |  |  |  |  |  |  |
| Growers |  |  |  |  |  |  |
| Growers |  |  |  |  |  |  |
| Piglets |  |  |  |  |  |  |
| Piglets |  |  |  |  |  |  |
| Piglets |  |  |  |  |  |  |

| **32. Comments** |
| --- |

| **33. Have you done any expansion in the pig enterprise since last visit?** |
| --- |

| ❑ | Yes |
| --- | --- |
| ❑ | No |

| **34. If yes, specify how:** |
| --- |

| **35. Do you keep records associated with the pig enterprise?** |
| --- |

| ❑ | Yes |
| --- | --- |
| ❑ | No |

| **36. What types of records?** |
| --- |

| ❑ | Feeds |
| --- | --- |
| ❑ | Reproduction and breeding |
| ❑ | Animal inventory (births, deaths, sales) |
| ❑ | Financial (income and expenditure) |
| ❑ | Other |

| If other, specify:__________________________________________ |
| --- |
|  |
|  |
|  |

| **37. Did you sell any pigs since the last visit?** |
| --- |

| ❑ | Yes |
| --- | --- |
| ❑ | No |

| **38. Indicate the numbers sold from each pig category:** |
| --- |

| Sales outlet: 1=Farm gate, 2=Village/local market. 3=Slaughterhouse/abbatoir, 4=Butchery, 5=Other (specify in coments) | | | | | |
| --- | --- | --- | --- | --- | --- |
|  |  |  |  |  |  |

|  | **How many sold** | **Weight (live)** | **Weight (carcass)** | **Price/head (UGX)** | **Sales outlet** |
| --- | --- | --- | --- | --- | --- |
| Breeding boars |  |  |  |  |  |
| Breeding sows |  |  |  |  |  |
| Growers |  |  |  |  |  |
| Piglets |  |  |  |  |  |

| **39. Comments** |
| --- |

| **40. Did you have any other income related to products from your own pigs since the last visit?** |
| --- |

| ❑ | Yes |
| --- | --- |
| ❑ | No |

| **41. If yes, what was the total income since the last visit?** |
| --- |

| ______________________________ |
| --- |

| **42. Do you own a breeding boar?**  **(If no skip to Q 46)** |
| --- |

| ❑ | Yes |
| --- | --- |
| ❑ | No |

| **43. Do you use it/them for own or communal breeding?** |
| --- |

| ❑ | Own |
| --- | --- |
| ❑ | Village/communal |
| ❑ | Other |

| If other, specify:___________________________________________ |
| --- |
| **44. How much do you charge per service (UGX or other)?** |

| _________________________________________________________________ |
| --- |

| **45. What was your total income from the breeding boar since the last visit?** |
| --- |

| ______________________________ |
| --- |

| **46. Indicate the source of breeding for the sows since the last visit** |
| --- |

| ❑ | Didnt do any breeding |
| --- | --- |
| ❑ | Own boar |
| ❑ | Other boar |

| If other, specify:__________________________________________ |
| --- |
|  |
| **47. What is the cost per service (UGX or other)?**_____________ |

| **48. What was your total expenditure for the breeding service since the last visit?** |
| --- |

| ______________________________ |
| --- |

| **49. Did you have any hired labour engaged in the pig enterprise since the last visit?** |
| --- |

| ❑ | Yes |
| --- | --- |
| ❑ | No |

| **50. If yes, what was your total expenditure for hired labour engaged in the pig enterprise since the last visit (UGX)?** |
| --- |

| ______________________________ |
| --- |

| **51. Did your pigs receive any medical treatments (deworming, antiparasitic, profylaxis, antibiotics, vaccination) since the last visit?** |
| --- |

| ❑ | Yes |
| --- | --- |
| ❑ | No |

| **52. If yes, what treatment(s)?** |
| --- |

| **53. What was your total expenditure for medical treatments since the last visit (UGX )?** |
| --- |

| ______________________________ |
| --- |

| **54. Did you have any expenditure for biosecurity equipment (protective clothing, boots, disinfectants etc) since the last visit?** |
| --- |

| ❑ | Yes |
| --- | --- |
| ❑ | No |

| **55. If yes, what sort of equipment did you buy?** |
| --- |

| ______________________________ |
| --- |
| **56. What was your total expenditure for bio security equipment since the last visit (UGX)?** |

| ______________________________ |
| --- |

| **57. Did you receive any extension service related to pigs since the last visit?** |
| --- |

| ❑ | Yes |
| --- | --- |
| ❑ | No |

| **58. What was your total expenditure for extension service related to pigs since the last visit (UGX)?** |
| --- |

| ______________________________ |
| --- |

| **59. What was your total expenditure on pig feeds since the last visit (UGX)?** |
| --- |

| ______________________________ |
| --- |

| **60. Since the last visit, did you have to sell any household assets due to losses incurred in the pig production?** |
| --- |

| ❑ | Yes |
| --- | --- |
| ❑ | No |

| **61. If yes, Indicate what asset and the price obtained** |
| --- |

|  | Type of asset | Price obtained |
| --- | --- | --- |
| Asset | ___________ | ___________ |
| Asset | ___________ | ___________ |
| Asset | ___________ | ___________ |

| **62. Since the last visit, how many times a week did your family eat meat (on average)?** |
| --- |

| ______________________________ |
| --- |

| **63. Have you needed any financial credit since the last visit?** |
| --- |

| ❑ | Yes |
| --- | --- |
| ❑ | No |

| **64. If yes, did you get the credit?** |
| --- |

| ❑ | Yes |
| --- | --- |
| ❑ | No |

| **65. If no, why was credit not acquired?** |
| --- |

| ❑ | No collateral |
| --- | --- |
| ❑ | Credit terms unfavourable |
| ❑ | Other |

| If other, specify:___________________________________________ |
| --- |
|  |
| **66. Amonut needed, recieved, interest rate and use of credit** |

| Reasons: 1=Family health problems, 2=Animal health problems, 3=Crop failure, 4=Investments, 5=Pay school fees, 6=Wedding, 7=Funeral, 8= Other, specify in comments  Use of credit: 1=Feeds, 2=Animal health, 3=Labour, 4=Capital costs, 5=Other (specify in comments) | | | | | |
| --- | --- | --- | --- | --- | --- |
|  |  |  |  |  |  |

|  | **Reason for needing credit** | **Amount needed** | **Amount received** | **Interest rate** | **Use of credit** |
| --- | --- | --- | --- | --- | --- |
| Credit 1 |  |  |  |  |  |
| Credit 2 |  |  |  |  |  |
| Credit 3 |  |  |  |  |  |

| **67. Comments** |
| --- |

| **68. Since the last visit, have you seen any wild pigs (warthogs or bushpigs) in your homestead or gardens?** |
| --- |

| ❑ | Yes |
| --- | --- |
| ❑ | No |

| If the respondent know, note which species:_____________________ |
| --- |

**69. Since the last visit;**

|  | No, none | Most not | Some yes, some not | Yes, most | Yes, all |
| --- | --- | --- | --- | --- | --- |
| Have the family been able to pay all needed school fees? |  |  |  |  |  |
| Have the family been able to meet all medical expenses that has come up |  |  |  |  |  |
| Have there been any family gatherings (weddings, funeral, baptisms) etc that had to be changed or postponed due to lack of money? |  |  |  |  |  |

| **70. Comments** |
| --- |
|  |

| **71. Since the last visit;** |
| --- |

|  | No, never | Most of the times not | Sometimes yes, sometimes not | Yes, most of the time | Yes, always |
| --- | --- | --- | --- | --- | --- |
| I feel more optimistic about the pig enterprise |  |  |  |  |  |
| There has been an increase in disputes, disagreements or jealousy among my neighbours |  |  |  |  |  |
| I have lost confidence in pig production |  |  |  |  |  |
| I am no longer participating in the social networks like I used to do |  |  |  |  |  |

| **72. Comments** |
| --- |
|  |

|  |
| --- |

| **73. How do you agree with the following statements;** |
| --- |

|  | Strongly disagree | Disagree | Neither agree nor disagree | Agree | Strongly agree |
| --- | --- | --- | --- | --- | --- |
| I think it is possible to protect my pigs from getting ASF by improving farm bio security |  |  |  |  |  |
| Eating pork from pigs that have died from ASF is safe for human health |  |  |  |  |  |
| If I would get a fair price I would be willing to sell all my healthy pigs when an ASF-outbreak were present in the area |  |  |  |  |  |
| I would like to invest in farm bio security if I received advice on what to do |  |  |  |  |  |
| I would be happy to buy pork products from a slaughterhouse that receive pigs that have been in contact with pigs dying from ASF |  |  |  |  |  |
| It is safe to give pigs water that has been used to clean knifes and pangas used for slaughtering and butchering as drinking water |  |  |  |  |  |
| Buying live pigs is a risk behaviour for contracting ASF |  |  |  |  |  |
| I don’t want to eat or buy pork from pigs that have died from ASF |  |  |  |  |  |
| I can not afford to invest in my pig farming |  |  |  |  |  |
| ASF can not be prevented |  |  |  |  |  |
| I can choose where/to whom I sell my pigs |  |  |  |  |  |
| Frequent selling and buying of pigs is necessary for successfull pig farming |  |  |  |  |  |
| Improved farm bio security improves pig health and pig growth |  |  |  |  |  |
| I could adopt my pig farming in order to have pigs ready for sale at specific times of the year |  |  |  |  |  |
| Cooking kills the ASF-virus |  |  |  |  |  |
| It is possible for me to tell visitors such as veterinarians, middle men and extension workers not to enter in the pig house with their own boots |  |  |  |  |  |
| If pork prices are lower in the neighbouring village due to them having an outbreak of ASF I will buy my pork there |  |  |  |  |  |

| **74. Comments** |
| --- |
