# Supplementary material for: Smallholders’ perceptions on biosecurity and disease control in relation to African swine fever in an endemically infected area in Northern Uganda
Source: BMC Vet Res. 2019 Aug 5;15:279. doi: 10.1186/s12917-019-2005-7 (PMC6683333; doi:10.1186/s12917-019-2005-7)
Supplement: Supplementary file 4 — Relevant associations between perceptions on general biosecurity, control interventions and attitudes to pig farming, and socio-economic variables and outbreaks of African swine fever (ASF) from a longitudinal interview study conducted with smallholder pig-farmers in northern Uganda 2014–2015. Perceptions were measured as agreements with given statements according to a Likert-scale with five levels: strongly agree = 5; agree = 4; neither agree nor disagree = 3; Disagree = 2; Strongly disagree = 1. Education level of the household head and spouse were recorded as a four-level categorical variable: no formal education =1; primary education =2; secondary education =3; higher level education =4. Associations were evaluated using the Kruskal Wallis test. Levels of significance is marked as * = p = 0.1; ** = p < 0.05; *** = p < 0.01. Associations with p-values > 0.1 are not displayed. Continuous explanatory variables are displayed as jittered scatter plots, and as Spearman rank colleration values, for categorical explanatory variables the median and mean value for each category are given. HH = household. (DOCX 91 kb) [file 12917_2019_2005_MOESM4_ESM.docx]

### Additional files 4:

Relevant associations between perceptions on general biosecurity, control interventions and attitudes to pig farming, and socio-economic variables and outbreaks of African swine fever (ASF) from a longitudinal interview study conducted with smallholder pig-farmers in northern Uganda 2014-2015. Perceptions were measured as agreements with given statements according to a Likert-scale with five levels: strongly agree=5; agree=4; neither agree nor disagree=3; Disagree=2; Strongly disagree=1. Education level of the household head and spouse were recorded as a four-level categorical variable: no formal education =1; primary education =2; secondary education =3; higher level education =4. Associations were evaluated using the Kruskal Wallis test. Levels of significance is marked as *=p=0.1; **=p<0.05; ***=p<0.01. Associations with p-values >0.1 are not displayed. Continuous explanatory variables are displayed as jittered scatter plots, and as Spearman rank correlation values, for categorical explanatory variables the median and mean value for each category are given. HH=household, ASF=African swine fever.

**4a) Second interview occasion, statements on general biosecurity**

| Statements/ variables | I think it is possible to protect my pigs from getting ASF by improving biosecurity | I would like to invest in biosecurity if I received advice | Improved biosecurity improves pig.health and growth | Eating pork from pigs that have died from ASF is safe for human health |
| --- | --- | --- | --- | --- |
| Continuous explanatory variables |  |  |  |  |
| Poverty score |  | 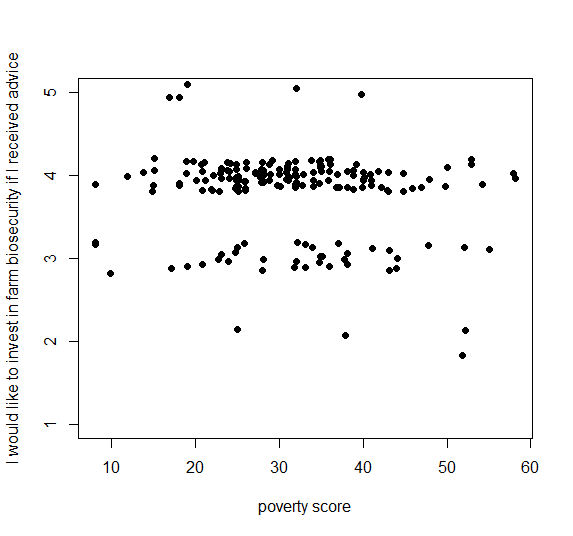 -0.12, ** |  |  |
| Number of pigs in HH |  |  |  | 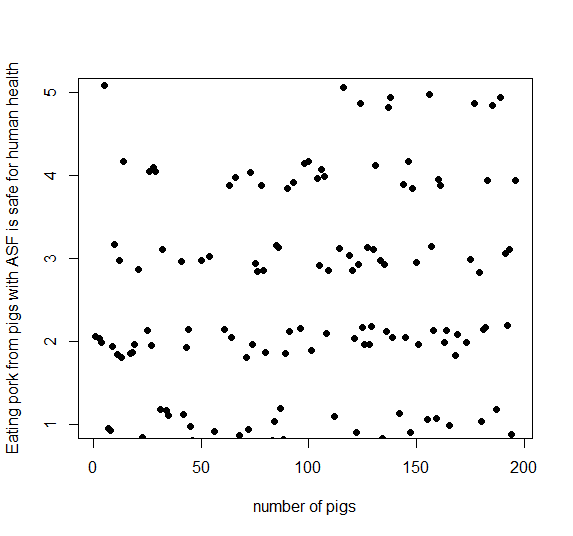  0.10, * |
| Categorical explanatory variables |  |  |  |  |
| Education level HH head |  |  |  |  |
| 1 |  |  |  | 3/3.17* |
| 2 |  |  |  | 3/2.69 |
| 3 |  |  |  | 2/2.49 |
| 4 |  |  |  | 2/2.38 |
| Education level spouse |  |  |  |  |
| 1 |  |  | 4/4.11** |  |
| 2 |  |  | 4/3.95 |  |
| 3 |  |  | 4/4.12 |  |
| 4 |  |  | 5/5.00 |  |
|  |  |  |  |  |
| ASF outbreak second interview occasion | 4/3.4** |  |  |  |
| No ASF outbreak second interview occasion | 4/3.82 |  |  |  |

**4b) Third interview occasion, statements on general biosecurity**

| Statements/  variables | I think it is possible to protect my pigs from getting ASF by improving biosecurity | I would like to invest in biosecurity if I receive advice | Eating pork from pigs that have died from ASF is safe for human health | I don’t want to eat or buy pork from pigs that have died from ASF |
| --- | --- | --- | --- | --- |
| Categorical explanatory variables |  |  |  |  |
| ASF outbreak third interview occasion | 4/3.43** | 4/3.53** | 3/3.04** | 4/3.60*** |
| No ASF outbreak third interview occasion | 4/3.84 | 4/3.84 | 2/2.60 | 5/4.54 |

**4c) Second interview occasion, statements on control interventions**

| Statements/ variables | I would be happy to buy pork products from a slaughterhouse  that pigs that have been in contact with pigs dying from ASF | I can choose where to/whom I sell my pigs |
| --- | --- | --- |
| Categorical explanatory variables |  |  |
| Education level spouse |  |  |
| 1 |  | 4/3.48** |
| 2 |  | 4/3.23 |
| 3 |  | 4/3.81 |
| 4 |  | 4/4.00 |
| ASF outbreak second interview occasion | 2/2.13** |  |
| No ASF outbreak second interview occasion | 2/1.71 |  |

**4d) Third interview occasion, statements on control interventions**

| Statements/ variables | If I would get a fair price I would be willing to sell all my healthy pigs when an ASF outbreak occoured in the area | I would be happy to buy pork products from a slaughterhouse that receive pigs that have been in contact with pigs dying from ASF | I could adopt my pig farming in order to have pigs ready for sale at specific times of the year |
| --- | --- | --- | --- |
| Continuous explanatory variables |  |  |  |
| Poverty score |  |  | 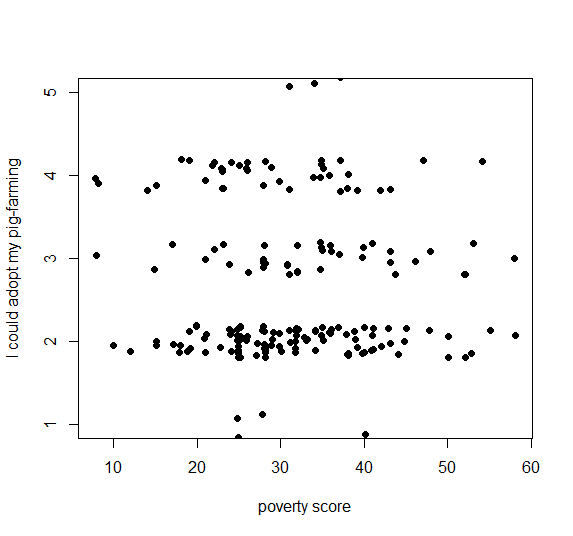  0, * |
| Categorical explanatory variables |  |  |  |
| ASF outbreak third interview occasion | 3/2.78** | 2/2.00** |  |
| No ASF outbreak third interview occasion | 2/2.37 | 2, 1.62 |  |

**4e)** **Second interview occasion, statements on attitudes**

| Statements/ variables | I have lost confidence in pig production |
| --- | --- |
| Continuous explanatory variables |  |
| Number of pigs in HH | 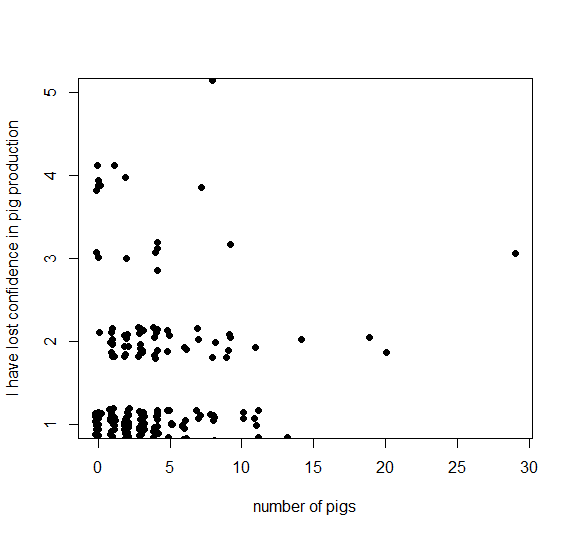 |
|  | 0.07, * |

**4f)** **Third interview occasion, statements on attitudes**

| Statements/ variables | I have lost confidence in pig production | There has been an increase in disputes, disagreements or jealousy among my neighbours |
| --- | --- | --- |
| Categorical explanatory variables |  |  |
| Education level HH head |  |  |
| 1 |  | 1/1.33** |
| 2 |  | 1/1.39 |
| 3 |  | 1/1.13 |
| 4 |  | 1/1.42 |
| ASF outbreak third interview occasion | 1/1.61** |  |
| No ASF outbreak third interview occasion | 1/1.32 |  |
